# Motif discovery in hospital ward vital signs observation networks

**DOI:** 10.1007/s13721-024-00490-1

**Published:** 2024-10-07

**Authors:** Rupert Ironside-Smith, Beryl Noë, Stuart M. Allen, Shannon Costello, Liam D. Turner

**Affiliations:** 1 School of Computer Science and Informatics, Cardiff University, Abacws, Senghennydd Road, Cardiff, CF24 4AG UK; 2 Florence Nightingale Faculty of Nursing, Midwifery and Palliative Care, King’s College London, 57 Waterloo Road, London, SE1 8WA UK

**Keywords:** Vital signs observations, Retrospective study, Network analysis, Subgraph ratio profile, Motif discovery

## Abstract

Vital signs observations are regular measurements used by healthcare staff to track a patient’s overall health status on hospital wards. We look at the potential in re-purposing aggregated and anonymised hospital data sources surrounding vital signs recording to provide new insights into how care is managed and delivered on wards. In this paper, we conduct a retrospective longitudinal observational study of 770,720 individual vital signs recordings across 20 hospital wards in South Wales (UK) and present a network modelling framework to explore and extract behavioural patterns via analysis of the resulting network structures at a global and local level. Self-loop edges, dyad, triad, and tetrad subgraphs were extracted and evaluated against a null model to determine individual statistical significance, and then combined into ward-level feature vectors to provide the means for determining notable behaviours across wards. Modelling data as a static network, by aggregating all vital sign observation data points, resulted in high uniformity but with the loss of important information which was better captured when modelling the static-temporal network, highlighting time’s crucial role as a network element. Wards mostly followed expected patterns, with chains or stand-alone supplementary observations by clinical staff. However, observation sequences that deviate from this are revealed in five identified motif subgraphs and 6 anti-motif subgraphs. External ward characteristics also showed minimal impact on the relative abundance of subgraphs, indicating a ‘superfamily’ phenomena that has been similarly seen in complex networks in other domains. Overall, the results show that network modelling effectively captured and exposed behaviours within vital signs observation data, and demonstrated uniformity across hospital wards in managing this practice.

## Introduction

Vital signs (e.g., blood pressure, heart rate, respiratory rate, temperature, level of consciousness, and oxygen saturation) are routinely recorded by healthcare staff in hospitals to track a patient’s overall health status. Individual vital sign scores are often combined into a single score as part of an Early Warning Score (EWS) system that measures vital signs across banded limits, such as NEWS-2 used within the UK (RCP 2012a). All patient vital signs recordings are required at regular observation intervals, which usually range between 15 min and 12 h, depending on the requirements of the ward. The propensity of certain observation intervals leads to clinical staff typically consolidating most routine patient observations into ‘ward rounds’ (ABUHB 2017) 2–4 times a day (Noë et al. 2022) in a notably non-uniform daily pattern of routine patient observations. It is also common that individual patient observation intervals are shortened as a cautionary response to threshold vital signs observations (Johnson et al. 2014) with respect to the relevant hospital policy (VitalPAC and ABUHB 2017). These observations typically happen hourly, but can be as short as 10–15 min in severe deterioration cases (e.g., sepsis onset, NICE (2023), or whenever continuous monitoring is not feasible).

Managing routine vital signs observations while supporting patients on individual intervals presents a complex challenge for clinical staff, reflected in documented compliance issues (i.e., when vital signs recordings are missed or delayed). The resulting variability in how routine vital signs observations are undertaken raises questions for staff on wards, hospital managers, and policymakers on whether wards run an appropriate operating schedule and how they support the delivery of care to patients on different observation intervals within it. This creates research motivations in needing to provide a basis to help quantify and identify patterns in ward behaviour. However, there are limited data sources available to help quantify and aggregate this, due to the cost and impracticalities of having either human observers on wards or installing bespoke new technologies. To address this in this study, we re-purpose existing data produced as a result of vital signs observations being undertaken as they are now commonly being recorded on mobile devices. A challenge exists however in appropriately modelling and summarising this activity. We propose a framework for modelling and analysing aggregated vital signs observation data as network graphs by considering the bed of the patient whose vital signs were measured as a node and generating a directed edge to the bed of the subsequent vital signs observation, if there is one (illustrated in Fig. 3).

Network modelling methods provide a versatile platform for understanding the structure and behaviour of complex, interconnected systems, and other aspects of healthcare systems have used these methods. For instance, network analysis was used to identify communities in hospital services (Niyirora and Aragones 2020) and which ones are the most central (Flemming et al. 2022). Other fields that have potential influencing factors on network structures (e.g., fixed locations and regular paths between these), such as airline (Verma et al. 2014; Jingyi and Yifang 2014) and road traffic networks (Cogoni et al. 2023; Logan and Goodwell 2023) have also been explored.

In such networks, it has been shown that local substructures can evolve over time (Agasse-Duval and Lawford 2018) and can be used to identify players that do not follow common behavioural patterns. For instance, Bounova (2009) showed in their airline case study that most airlines keep to a typical ‘hub and spoke’ structure, whilst the Southwest airline operates with an unusually random flight pattern. Tracking patterns of substructure growth suggests however that Southwest has become more centralised, closer to the typical hub-spoke topologies of other airlines. In the context of a hospital, identifying typical ward operating behaviour, and therefore recognising wards that operate atypically, or have done so for certain periods, is critical information for hospital stakeholders as it may highlight important implications such as under-resourcing, but also may be indicative of the ward architecture and environment, or other aspects of staff management. This can then provide the basis for stakeholders to observe changes as a result of any policy, training, or management adjustments using patient vital signs observation patterns, ultimately helping to improve overall ward efficiency and patient care.

Using this framework, we model a large dataset of vital sign recordings across 20 hospital wards spread across multiple hospitals using different network representations and explore how behaviours can manifest in the network representation through analysing their inherent structures. In particular, we look to the relative frequency of highly recurrent substructure patterns (Milo et al. 2002, 2004a) within vital signs observation sequences in comparison to what may occur randomly as this has been shown to carry significant information about the given network’s function (Vázquez et al. 2004) without being influenced by mediating factors (such as size, specialism, etc). We also explore the similarities and differences within and across wards using these structures and how they can characterise the management of care in ward environments. We summarise our research motivations of this study using the following research questions:

**RQ1**: Can vital signs observation sequences be effectively described using network modelling and analysis methods to reveal behaviours in how care is managed?
**RQ2**: What specific local structures within network representations of vital signs observation sequences can be identified as motifs, what are their relative significance, and what could they represent in how care is managed?
**RQ3**: To what extent are networks derived from aggregated vital signs observations data individualised to wards, or do they exhibit similarities?

Through the modelling and analyses performed to answer these questions, we reveal new evidence of ward heuristics being used to help manage patient care, providing notable cross-disciplinary contributions:

1. A novel framework for modelling sequences of patient vital sign observation recordings as a network representation, and a discussion on how this can be flexible and scale to support broader applied applications;
2. The identification of ‘typical’ behaviours in vital sign observation sequences and a discussion of how this may be influenced by overarching care policies;
3. An evaluation of highly regular and irregular network substructures, and how these combine to create a common network signature that is unique to hospital wards.

### Paper outline

**Fig. 1:**
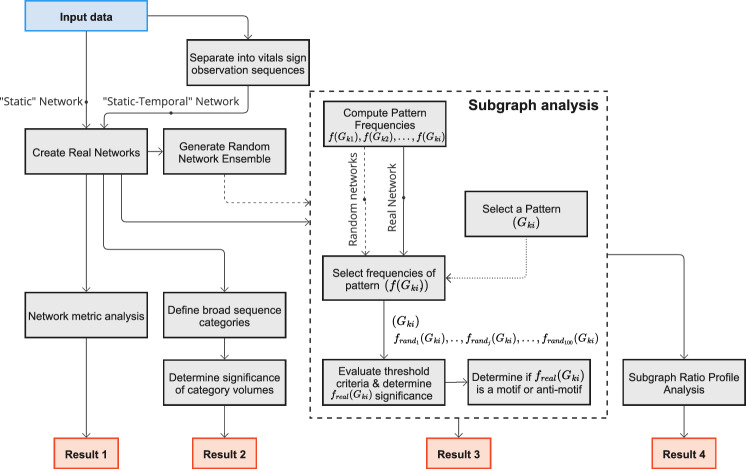
Flow diagram of study methodology

This study is centred around modelling vital sign observations using different network representations and analyses the frequency of regular repeating isomorphic patterns, known as ‘subgraphs’, using appropriate statistical significance measures, to identify those that occur more or less frequently than would be explained by random chance. The study outline is described in Fig. 1 and contributes 4 key results that describe vital sign observation management heuristics in ward environments based on the networks and their local structures.

The remainder of the paper is organised as outlined below.

The Related Works section supplements the research motivations in the Introduction by discussing key related studies and identifying relevant research gaps that have informed our research questions. The Materials and Methods section then introduces the content of our dataset and the framework for modelling and analysing the data, including how we define two network models per ward appropriately from the dataset: one representation that aggregates all data points for the period and one that is defined by an additional temporal dimension. We continue with an explanation of how subgraph frequencies are counted and discuss which subgraphs are considered in this study. Next, we describe minimum probability, frequency, and distribution criteria that individual subgraph patterns will be tested against to determine whether they are representative of regular network behaviour. We follow by describing a statistical measure of relative abundance to determine their strength. Finally, we also describe the construction of a ‘null model’, to which the subgraph frequencies are compared against.

We begin our Results section by exploring the global structure of the networks using different network analysis measures (e.g., density, clustering, and closeness) and how the topological structure changes with the addition of a temporal dimension. This is followed by categorising networks with a temporal element using high-level features to assess the consistency of observation sequence management between wards. After considering these broad features, we assess the local structure of the networks using subgraph analysis to observe how well represented specific sub-structures are relative to random networks (i.e., high relative significance), and consider their place as a network ‘motif’ (Milo et al. 2002; Ashford et al. 2019). Finally, we use subgraph significance scores to construct fixed-length feature vectors for each individual ward, where the length is equal to the number of considered subgraphs. This functional representation of individual ward network topological structures can be used to determine the presence and strength of a Subgraph Ratio Profile grouping (Milo et al. 2004a; Felmlee et al. 2021).

This is followed by the Discussion section, which examines the implications of the results against the research questions, as well as additional clinical implications. After this, the limitations of the study are discussed, along with suggestions for future work, including potential applications with machine learning. The paper concludes with a summary of the study and its contributions, as well as advantages and disadvantages of the proposed framework.

## Related work

Traditionally, EWS systems have required manual calculation of total parameter scores and documentation on bedside paper charts, like the NHS’ NEWS2 vital signs observations chart (RCP 2012b). However, a significant portion of secondary care is undergoing a transition to electronic documentation using handheld mobile devices (NICE 2020), such as in the UK through the NHS Long Term Plan (NHS 2019).

Introducing electronic documentation and tracking for patient vital signs observations (also known as ‘e-observations’) has been shown to have improvements in documentation quality (Wong et al. 2017; Prytherch et al. 2006; Cardona-Morrell et al. 2016; Downey et al. 2017; Ludikhuize et al. 2012), more time attributed to patient care (Gyi et al. 2019; Mohammed et al. 2009; Kolic et al. 2015), and improved timeliness compliance (Gale-Grant and Quist 2018). It has also provided a basis for studies to retrospectively examine the data resulting from vital sign observations.

**Fig. 2:**
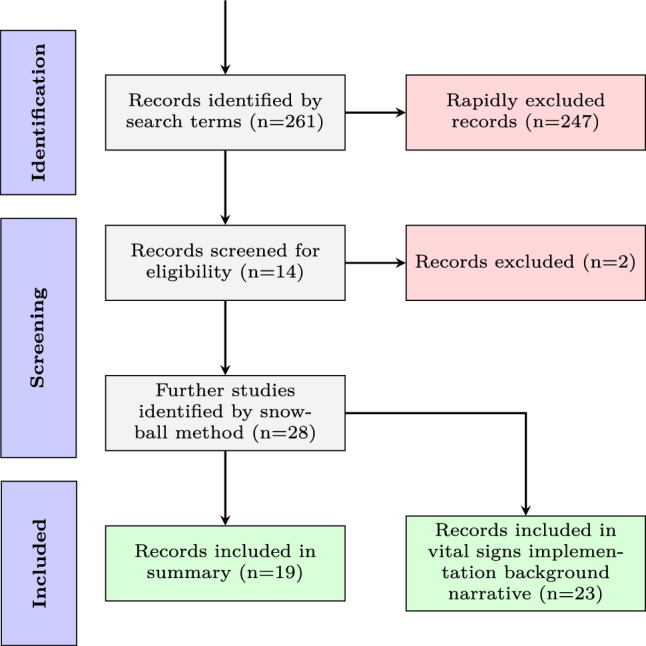
PRISMA flow diagram of literature search methodology

In this section, we outline our search strategy (Fig. 2), including key terms and selection criteria (Table 1) used to identify relevant studies. We summarise all retrospective studies on vital sign observations that present ward-level outcomes in Table 11. We then discuss the significant impact of e-observations, highlighting how their implementation has improved patient care and established a new field of study focused on the frequency and documentation compliance of vital sign observations. Following this, we consider the application of complex networks in various case examples for modelling and analysing human behaviour. We also discuss the usefulness of studying the topological structure and relative frequencies of subgraphs in understanding abstract and often obscured network behaviours, and how the combination of relative subgraph frequencies can facilitate a holistic comparison to other networks, including those of different types. Finally, we draw on the research gaps identified across these areas and discuss how they have informed the research questions and methodology used.

**Table 1:** Inclusion and exclusion criteria for literature review

Inclusion criteria	Exclusion criteria
The paper is in English	The paper is not in English
Peer reviewed journal or conference publication	Other publication types, such as editorials, letters, legal cases, and interviews
A retrospective longitudinal observational study of a vital sign observation dataset	Qualitative or survey studies
Discusses outcomes of ward-level vital sign observation management features (such as frequency and compliance to hospital policy)	Discusses patient outcomes or EWS effectiveness
Full text is available	No full text available

### Search strategy

Due to the variability in datasets, study settings, and periods in this field, this study completed a scoping review to identify relevant publications that describe retrospective studies on a vital signs observations. Figure 2 reports the flow chart of the study selection, which begins with a literature search in the bibliographic databases *PubMed* and *Google Scholar* (title, abstract, keywords) using the following search terms:

*“Vital signs”* + *“retrospective”* + (*“missed”* or *“VitalPAC”*)

and the following related search terms: *“vital signs”*, *“Retrospective”*, *Late*, *“CareFlow”*, *“e-obs”*, *“e-observations”*, *“electronic health record”*. EndNote 20 referencing software (Clarivate 2013) was used to screen titles and abstracts of returned studies and define a core subset of highly-cited studies that undertake a retrospective observational study on vital signs observation data. Subsequently, we used the ‘snowball’ search method (Wohlin 2014) to identify additional works focused on ward-level outcomes in response to the procedure of undertaking vital sign observations (e.g., frequency and compliance to hospital policies). We excluded records that focused on individual patient outcome (e.g., mortality rates) and records that considered the predicative capabilities of vital signs when they are used as part of an EWS score. Relevant studies that discussed the implementation and handling of vital sign observation data inputting methods, such as VitalPAC, are included.

### Analysis of vital signs observations

The literature search highlights that most retrospective studies on vital signs observation datasets are primarily focused on patient outcomes, however, we identified 19 studies that meet the inclusion criteria described in Table 1. The dataset, study setting, period, purpose, and results for all included papers are summarised in Table 11. Among these 19 studies, 10 studies discussed both the frequency and compliance of documenting a complete set of vital signs in adherence to hospital scheduling policy, 7 only considered compliance, and the last 2, only examined the frequency. It is clear from this overview that the literature discussing ward-level outcomes that arise from different vital sign observation management practices is still limited.

Shortcomings in the frequency and compliance of patient observations have been identified (Leuvan and Mitchell 2008; Johnson et al. 2014; van Galen et al. 2016; Gale-Grant and Quist 2018; Eddahchouri et al. 2021; Jackson et al. 2023) and have partially been attributed to staff interaction with e-observation systems (Miltner et al. 2014; Watson et al. 2014) and the impact of staffing levels or shift lengths on documentation compliance and timeliness (Armstrong et al. 2008; Dall’Ora 2017; Griffiths et al. 2018; Redfern et al. 2019; Dall’Ora et al. 2019; Smith et al. 2020). Additionally, studies have examined how the hourly volume of observations changes throughout the day (McGain et al. 2008; Hands et al. 2013), and whether inter-wards differences (Noë et al. 2022) or notable periods (Kostakis et al. 2021) can be identified. However, there has been limited consideration of the potential impacts of the sequence in which observations are undertaken, which informs the design of the modelling and analysis framework in the study.

E-observations of vital signs have been suggested to not only provide a practical and affordable clinical improvement (Gale-Grant and Quist 2018), but also opened new frontiers for inter-ward patient management analysis (Dall’Ora et al. 2020; Griffiths et al. 2018) and the consideration of new patient management metrics, such as timeliness and compliance (Watson et al. 2014). Wards have been shown to broadly align in daily observation volume distribution (i.e., typical ward round times) when categorised by observation interval distributions (Kostakis et al. 2021; Noë et al. 2022). A method to stratify vital signs observation timeliness with respect to the Time To Next Observation (TTNO) was also defined by the Missed Care Study Group (Griffiths et al. 2018). This has led to further findings in the space, including highlighting that shorter observation intervals and high NEWS patients have been shown to have the most vital sign observation omissions (Oliveira et al. 2022; Kostakis et al. 2021; Redfern et al. 2019).

Despite the merits of e-observations, there has been evidence of data consistency shortcomings and poor device implementation that can encourage nurse workarounds using traditional methods (RCP 2012a; Yeung et al. 2012), particularly when the ability to record legitimate reasons for missing observations is often omitted in the software (Hope et al. 2019). So far, works utilising e-observations datasets have been predominantly patient-focused, with most attention directed to evaluating EWS efficacy (e.g., Kellett 2011; Bleyer et al. 2011), but a few studies have corroborated the timeliness of vital signs observations to clinical staff management factors such as staffing levels (Griffiths et al. 2016; Redfern et al. 2019) and shift length (Dall’Ora et al. 2019) of registered nurses.

Other studies have also touched on well-established understandings of general intra-patient observation management behaviours, such as how vital signs observations are consolidated into ward rounds (Hands et al. 2013), and how this practice changes when operating in different periods (such as COVID-19, Kostakis et al. 2021) or ward specialism (Noë et al. 2022). However, a notable gap exists across these studies in them having limited granularity in the behaviours exposed beyond the grouping into ward rounds). How vital-sign observations are being undertaken and prioritised from patient to patient at a higher granularity forms the basis for the research methodology in this study.

### Analysis of complex networks

Network modelling approaches have been used as a means to model and analyse complex systems. This includes analysis within the healthcare domain where network analysis methods have been used to identify communities in hospital services (Niyirora and Aragones 2020), and which ones are the most central (Flemming et al. 2022). However, to the best of our knowledge, network modelling has not been used to model and examine human behaviour in the healthcare domain, in a similar manner to other domains such as aircraft (Agasse-Duval and Lawford 2018), or human mobility networks more broadly (Hossmann et al. 2011). At a high level, this research gap has informed the overarching methodology of this study.

#### Network representations

There are further considerations in how the network models can be constructed and analysed. The network representation of vital signs observations is expected to be largely influenced by common patient management procedures (such as deteriorating patient policies ABUHB 2017) that may dictate the recurrence of specific network features (Alon 2003), much like how transportation links (Pellegrini et al. 2020) are physically constrained by roads. Because of this, ward operating policy will act as a key point of reference when contextualising the resulting network structures.

Static networks using simple graphs have been shown to be effective in representing key information on relationships between entities in complex systems (Milo et al. 2004b). Therefore, due to the limited literature surrounding representations of vital sign observation sequences, static networks inform a basis of the methodology in this study. However, as it is unclear to what extent modelling vital signs observation networks statically obscures dynamic ward-level behaviours and the possible causes behind them (Tantipathananandh et al. 2007), other network representations integrating a temporal element are also used and provide a basis for comparison. By considering the sequence in which edges occur, it is envisaged that this may provide a richer context to understand ward-management behaviour and determine interactions which occur simultaneously.

The ‘temporal’ network dimension brings additional challenges that distinguish it from static networks, and can be characterised in a number of ways. Existing methods either consider a ‘static-temporal’ network representation by modelling networks as strictly growing where a pair of nodes connect once and stay connected forever (Leskovec et al. 2007; Jacobs et al. 2015) or where a series of static network snapshots are taken at sequential moments in time (Tantipathananandh et al. 2007; Mucha et al. 2010; Hulovatyy and Milenkovic 2015). Alternatively, a strictly ‘temporal network’ representation (Cinaglia and Cannataro 2022; Tu et al. 2018b) may also be considered, where the network is defined by a set of nodes and a collection of directed temporal edges with a timestamp on each edge (Viswanath et al. 2009; Paranjape et al. 2016). For this study, we compare the static network against a static-temporal network for which snapshots are defined by heuristic ward-level behaviour: ward rounds. Although this method has been suggested to overlook the continuity of dynamic systems by discarding the relationship between each snapshot (Holme and Saramäki 2012) and thereby potentially limiting the ability to capture changes at a finer granularity (Paranjape et al. 2016; Tu et al. 2018b), it has also been suggested that static networks are more precisely modelled when integrating this form of temporal data (Chen et al. 2013).

#### Network analysis and comparison

For the analysis of the different network representations, summative statistics of the global structure (e.g., density, degree centrality, and clustering coefficients) have widely been used to contextualise the properties of complex networks. These metrics do not only offer the potential to characterise a wide range of natural phenomena and human behaviour patterns but have also demonstrated correlations with measures of more local substructures within the network (e.g., Vázquez et al. 2004; Turner et al. 2019).

Analysis of the sub-structures within networks using various subgraph analysis methods (Jazayeri and Yang 2020) are adopted in increasing more fields since their original applications to biological networks (Milo et al. 2002; Shen-Orr et al. 2002). This has included analysis of Wikipedia articles (Zlati et al. 2006) and editor behaviour (Wu et al. 2011) interactions, as well as investigating the drivers behind network functions, such as identifying users involved in YouTube ad spam campaigns (O’Callaghan et al. 2021). This motivates addressing a further research gap in applying global and local network analysis methods to a new domain in this study, i.e., in network representations of vital signs observation data. Within subgraph analysis, a key consideration is the size of the subgraphs. Whilst many studies focus on particular types of subgraphs, the most common being triads ($V=3$, where *V* is the total vertices in the graph) (e.g., Milo et al. 2004b; Ashford et al. 2019; Turner et al. 2019), others also include other degrees, such as dyads ($V=2$) and/or tetrads ($V=4$) (e.g., (Felmlee et al. 2021; Tu et al. 2020b). Exploring subgraph groups together (e.g., both triads and tetrads) will provide the scope to determine the suitability of each in effectively capturing the nuances of network behaviours whilst ensuring robustness against potential different higher-order clustering (Milo et al. 2004b; Olaf 2004; Benson et al. 2016; Agasse-Duval and Lawford 2018).

Importantly, the consideration of network self-loop edges is also underrepresented in subgraph analysis of complex networks, but they are also often missing in the networks analysed (e.g., social networks or protein structures) or may not be of interest, except in some demonstrated cases (Becskei and Serrano 2000; Nitzan Rosenfeld and Alon 2002). However, this is a key consideration for vital sign observations, where multiple repeat observations could be undertaken with a patient over a short period of time before the observation of another patient. A notable research gap exists in developing a framework for considering a range of subgraph types along with self-loops that addresses the limitations of existing approaches (e.g., gl2vec (Tu et al. 2020b)) that can be used to identify notable motifs in the network structure as well as serve as a richer basis for comparison across networks.

Various approaches have been proposed for counting the frequency of the chosen types of subgraphs or motifs (Jazayeri and Yang 2020). Within this, different approaches aim to address different challenges that can arise in the mining and counting process. For example, very large networks can cause the process to become too computationally expensive and too slow to be practical. This has resulted in various studies proposing the estimation of subgraph frequency counting, rather than exact counts (Lotito et al. 2024). This can include the adoption of machine learning in the methodology, such as Graph Neural Networks (e.g., Besta et al. 2022; Kanatsoulis and Ribeiro 2024). However, while these methods offer potential speed advantages, the disadvantages in potential inaccuracies should be considered with the application domain. In the context of this study, the network representations are relatively small as a result of the physical and environmental constraints of individual hospitals and wards with limited number of staff and beds. This knowledge combined with the context of safety in a healthcare domain motivates the use of exact subgraph counting in examining the current dataset. The existence of these methods however motivate a design consideration for our proposed framework in enabling flexibility for alternative counting methods (i.e. estimation) for future works.

Furthermore, assessing whether a selection of networks exhibit distinct or similar behaviours through global or local network measures (e.g., subgraph frequency analysis) can be challenging due to lack of comparable references. Milo et al. (2004a) presented an approach for comparing network substructures via a normalised feature vector of significance metrics (usually z-score or relative abundance ($\Delta$)) for all considered subgraphs. This is known as a Subgraph Ratio Profile (SRP), or Triad Subgraph Profile (TSP). Subgraph significance metrics are typically derived from subgraph abundance rates in the study network in comparison to subgraph appearance rates in an appropriate sample of equivalent randomized networks (e.g., of the same size and degree sequence; Artzy-Randrup et al. 2004; Milenković et al. 2009), called a “null model” (Wasserman and Faust 1994). This method facilitates comparison between numerous networks and is insensitive to network size and degree (Tu et al. 2020a), and as such, has become an established method. SRP analysis has demonstrated unique profile groups across different networks such as the World Wide Web, Wikipedia articles, and global energy trade (Milo et al. 2004a; Zlati et al. 2006; Shutters et al. 2022). Additionally, SRP analysis has been applied for clustering similar networks (Ashford et al. 2019), tracking evolutionary changes in biological networks (Kashtan and Alon 2005), and serving as a vector for feature representation (Tu et al. 2020b), thereby enabling comparisons with various machine learning analysis methods.

### Summary of research gaps

The research gaps identified that will inform the methodology of this study can be broadly summarised under several key areas. Firstly, as discussed in Sect. 2.2, previous studies have not explicitly modelled vital sign observations at a high granularity (e.g, sequences from one observation to the next). Most studies focus on patient outcomes, such as the effectiveness of EWS packages or the compliance with hospital documentation-practice policies. It is envisaged that the contributions of this study will complement these works by providing insight on ward behaviour at a higher granularity and from an adjacent perspective.

Secondly, as discussed in Sects. 2.3 and 2.3.1, while network representations and analysis has been used to model and explore a wide range of complex systems, including human behaviour in different domains, there has been limited use of modelling human behaviour in providing healthcare. Previous studies have shown how distinct behaviours can manifest in network representations of independent players within different case-study domains (e.g., social networks, road networks), and an opportunity exists to explore how distinct vital observation networks structures are for different wards and hospitals.

Thirdly, as discussed in Sect. 2.3.2 existing cross-disciplinary studies that do draw on network modelling techniques often focus on a single group of subgraphs (e.g. triads) and also omit or under-represent self-loop edges. A feature of the vital signs observation data is that doing so would overlook key behaviours, such as a clinical staff member rapidly repeating a vital sign observation, or the impact of architectural features in wards (i.e., if rooms have one, two, or four beds). This would have a notable effect on practical applications in what could be effectively derived from wards and for comparisons within and across them. There is therefore an opportunity alongside the exploration of the dataset for an extended representation of different types of subgraphs in the motif discovery and comparison methodology and analysis that could also be used more generally beyond this study in the analysis of networks.

## Materials and methods

This study employs a vital signs observations dataset spanning 20 wards from various hospital sites and different specialisms. Utilising this dataset, we present a framework that models this data through multiple network representations with an objective to analysis key properties of their structure and facilitate comparison of the similarities and differences in the behaviour surrounding the management of patient care.

### Data overview

**Table 2:** A simplified example set of vital signs observations recordings for a single ward

Observation	Time	Staff	Patient	Bed	NEWS	Concerned?
1	10:00	1	A	1	1	No
2	10:04	1	B	2	3	No
3	10:09	1	C	3	1	No
4	10:14	1	B	2	4	No
5	10:23	1	D	4	4	No
6	10:31	2	D	4	3	No
7	12:45	1	E	5	1	No

Example NEWS and ‘Concerned?’ scores are contextual for scope of further analysis

In this study, we examine a large dataset of 770,720 individual vital sign observations, for which we define an ‘vital sign observation’ as a 7-dimensional vector that includes: observation number, time of observation, staff ID, patient ID, bed ID, NEWS, and staff concern (a checkbox, optionally selected at the bedside, indicating either ‘yes’ or the default ‘no’). We describe an example set of 7 anonymised vital signs observations in Table 2. This study spans 20 different wards of 8 different specialisations (medical, surgical, rehabilitation, care of the elderly, orthopaedic, cardiology, and acute stroke) from 7 different hospital sites run by the Aneurin Bevan University Health Board (ABUHB) in South Wales, UK (see Table 3). The selected wards have a consistent framework of e-observations and staff have received substantiated e-observations training. Additionally, the timeframes were chosen such as that e-observations were consistently recorded for a full year (4 wards for 2019 and 16 for 2022). The dataset is compiled from CareFlow eObservations,^1^ a software installed on mobile devices on wards (e.g., iPod Touch, or an equivalent small tablet) which, among other features, allow vital signs data to be entered at the bedside. CareFlow Vitals also performs automatic NEWS scoring and observation-interval calculations (ABUHB 2017).

**Table 3:** Summary of characteristics for each of the 20 study wards

Ward	Year	Ward type	Total observations	Staff IDs	Patient IDs	Bed IDs
W1	2019	Medical	44,573	183	904	32
W2	2019	Medical	47,533	129	1396	30
W3	2019	Surgical	44,837	251	1335	32
W4	2019	Surgical	42,356	226	1572	32
W5	2022	Gastroenterology	54,271	166	1403	31
W6	2022	Respiratory	45,515	184	1606	31
W7	2022	Medical	37,718	151	308	30
W8	2022	Medical	25,302	161	404	32
W9	2022	Cardiology	22,155	87	735	17
W10	2022	Rehabilitation	25,445	169	453	32
W11	2022	Medical	28,501	137	422	30
W12	2022	Care Of The Elderly	39,245	116	651	30
W13	2022	Rehabilitation	31,326	132	224	25
W14	2022	Cardiology	35,203	141	1352	24
W15	2022	Trauma & Orthopaedics	30,429	218	1267	28
W16	2022	Trauma & Orthopaedics	25,910	79	1764	35
W17	2022	Rehabilitation	11,970	100	178	18
W18	2022	Rehabilitation	28,408	53	233	34
W19	2022	Medical	9294	40	118	12
W20	2022	Rehabilitation	24,977	147	543	30

### Static network construction

**Fig. 3:**
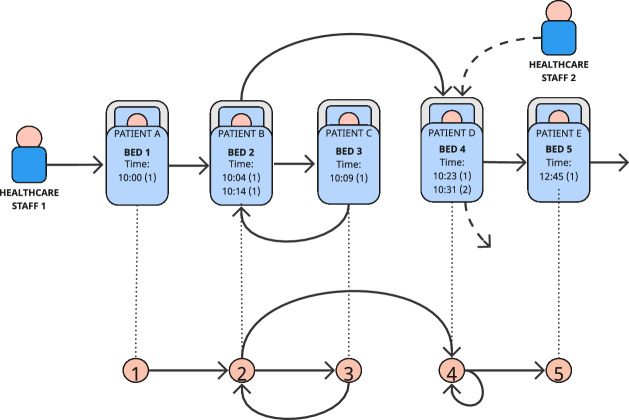
Visual representation of the example set of vital signs observations (Table 2) and its corresponding network representation

To the best of our knowledge, no-one has yet represented a vital sign observation dataset as a network of clinical staff movement patterns. Let the vital sign observation network of a given ward be defined by $G=(V, E)$, where each bed is represented by a node v $\in$ V. To maintain continuity between observation recordings, nodes were chosen to represent bed IDs rather than patient IDs, since patients may move during their stay, whereas bed configurations are largely consistent and therefore a more accurate reflection of regular ward actions. Directed edges are added to the network by iterating over observation records in ascending time order. An edge ($v_{i}$, $v_{j}$) $\in V$ indicates that an observation has taken place in the bed $v_{j}$ directly after an observation in bed $v_{i}$. We allow self-loops ($v_{i}$, $v_{i}$), but not multi-edges (where the same bed sequences can occur multiple times). This is illustrated in Fig. 3.

### Subgraph discovery and time complexity

**Fig. 4:**
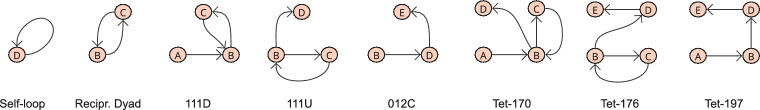
Triads and tetrads represented in the example network shown in Fig. 3, where the full catalogue of triads is demonstrated in Fig. 13 and the full catalogue of Tetrads is demonstrated in Fig. 14

The results of this study are determined by the frequencies of induced directed subgraphs of *V* nodes, i.e., $V=2$ (dyads), $V=3$ (triads), $V=4$ (tetrads), and self-loop edges. We treat self-loop edges as ‘single-node network motifs’ to reduce the huge number of higher-order subgraph permutations that would need to be considered otherwise (i.e., for each triad subgraph, 7 self-loop variants would also be possible, and 15 for each tetrad subgraph), therefore reducing analysis complexity (Nitzan Rosenfeld and Alon 2002) and noise (Becskei and Serrano 2000). The broad range of subgraph degree sequences will ensure robustness when identifying superfamily behaviour and provide more leverage to expose different organisational patterns between wards (Milo et al. 2004b; Benson et al. 2016). We also follow the common practice to reduce the 16 possible isomorphic directed triads to the 13 weakly connected triads (Tu et al. 2018a; Felmlee et al. 2021), excluding those that do not include all three nodes (003, 012, 102, see Fig. 13) and evaluate a dyad representation (described as triads 012 and 102 in Fig. 13) separately (Benson et al. 2016). The same practice is used for tetrads (e.g., Kashtan and Alon 2005; Krumov et al. 2011; Shen-Orr et al. 2002), for which we use a sample of the 199 weakly connected directed tetrads (McMillan and Felmlee 2020), (Fig. 4)..

This study utilises two tools for the discovery of network subgraphs of degrees $V=1$ to $V=4$:

- NetworkX Triadic Census^2^ for subgraph instances of degree sequences $V=1$, $V=2$, and $V=3$ (see Fig. 13).
- gTrieScanner^3^ for subgraph instances for degree sequence $V=4$ (see Fig. 14).

We utilise two methods for this study because of the diverse nature of subgraphs that are considered. The Triadic census program does not count subgraphs of greater degree than $V=3$, and gTrieScanner does not support counting dyad and self loop edges, which are also essential components of this study. The NetworkX triadic census sub-quadratic algorithm suggests a time complexity *O*(|*E*|), where *E* is the number of edges in the network graph, to record subgraph frequencies for all directed subgraphs of degree sequences $V=1$, $V=2$, and $V=3$ in ’large’ and ’sparse’ networks. In other cases, the program uses an algorithm of quadratic complexity $O(|V|^2)$, where *V* is the number of vertices (Batagelj and Mrvar 2001; Moody 1998). Our networks are typically small, where $V=32$ in our largest wards, so even in a connected graph representation we expect low latency. The time complexity for defining a census of degree *n* subgraphs using a gTrie data structure method in practical applications is effectively managed by the structure of the network graph. Ribeiro and Silva (2010) suggests that their gTrie algorithm performs worst for dense graphs with deep trees, however, the method still outperformed alternative algorithms. For our application of moderately sized graphs and relatively small subgraph patterns, we can expect low latency for defining subgraph censuses. We also considered using machine learning for probabilistic network motif mining, with the main advantage being speed, though it comes with the trade-off of being an approximation (Ribeiro et al. 2021; Oliver et al. 2022). In the context of this study and the relatively small network sizes, exact subgraph counting and motif extraction is considered suitable. However, the methodology here is flexible for any future applications that would have significantly larger networks.

### Static-temporal network construction

**Fig. 5:**
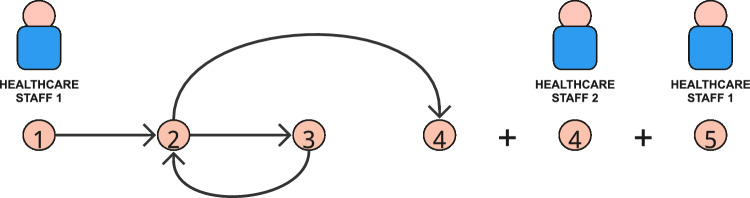
Adjusted network representation of the example network in Fig. 3. The network is segregated into 3; two ’stand-alone’ observations, one by Healthcare Staff 1 and one by Healthcare Staff 2, and one that can be described as an ’immediate intermediate patient return’ by Healthcare Staff 1

**Fig. 6:**
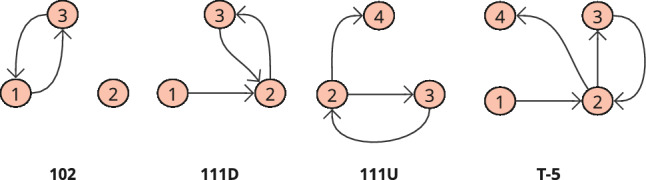
Clinical staff action in the static-temporal networks illustrated in Fig. 5 can be represented by four subgraphs; a reciprocating dyad, two triads, (111D and 111U, Fig. 13), and one tetrad (T5, Fig. 14)

We also consider including temporality by building additional network representations from sequences of vital signs observations undertaken in quick succession by individual clinical members of staff. We note that the number of patients observed within a staff ward round is highly varied and non-standard, from regular single patient entries (illustrated by Healthcare Staff 2 in Fig. 3) up to as many as 20 vital sign observations long. We consider each temporally sequential ward round as a static network ‘snapshot’ that is evaluated individually, before aggregating the results for ward-level comparisons. Figure 5 illustrates how the example vital signs observation recordings in Table 2 would be represented as three isolated network components, and Fig. 6 highlights the extracted subgraph patterns.

CareFlow e-Observations provide no predefined labelling for when a vital sign observation has been completed as part of a ward round. To our knowledge, the current literature has also not discussed any formal definition as to what can be considered a consistent start and end point of different ward rounds. We therefore define that when a clinical staff member is no longer undertaking vital signs observations in ‘quick succession’ as the end point of a ward round, where the time between observations exceeds a specified $\partial$, in minutes. $\partial$ must be inclusive of short additional duties that occur within rounds (e.g., escalating a patient to a senior staff member or assisting with a patient comfort break before returning to the round), yet exclude other core duties (e.g., washing, clinical investigations, handovers, drug rounds, ward-transfers and mealtimes) (Hands et al. 2013). Additionally, $\partial$ should be calculated on a per-ward basis as architecture, staffing levels, and time to complete observations differ significantly by ward size and type. However, $\partial$ should also be small enough to avoid incorporating the start of subsequent observation sequences or stand-alone observations (such as hourly patients) to not over-inflate volumes of complex observation sequences. Therefore, it is practical to calculate $\partial$ for a specific ward by utilising the function for the time taken to ‘miss’ a patient’s vital signs observation (e.g., $2\times$ observation interval $+$ observation interval, Noë et al. 2022), based on the shortest observation interval administered to a patient in that ward over the entire study period. In practice the shortest patient observation interval varies between wards, typically being 10 or 15 min, thus $\partial$ is usually 30 or 45 min.

### Statistical analysis

This analysis is rooted in statistical methods to identify subgraphs that occur dis-proportionally frequently to those seen in a null model, indicating that they may represent likely network motifs or anti-motifs. We use these to establish similarities and differences in how wards operate and to identify key behaviours that are abundant and those that are uncommon.

#### Null-model network construction

We construct our null model as the average of 100 random networks that are generated as similar as possible to the original dataset. This ensures statistical meaning in the results (Milo et al. 2004a; Ribeiro et al. 2009), avoids biased estimates of subgraph presence in the case of highly skewed degree sequences (Artzy-Randrup et al. 2004), and prevents an overly well-rounded significance profile as a result of the noise in the data. To achieve this, we additionally control the distribution of in-degree and out-degree vertices to node and edge volumes, known as a Bi-Degree Edge (BDE, Milo et al. 2004a; Shen-Orr et al. 2002; Kashtan and Alon 2005; Yeger-Lotem 2004) or Bi-Degree Sequence (BDS, Tu et al. 2020a) model. BDE models are typically appropriate for networks whose connectivity distribution differs markedly from that of a random graph with uniformly distributed links (Berg and Lässig 2004). Each randomly generated network is intended to represent a random staff path through a virtual ward (like the example paths illustrated in Fig. 3. Individual clinical staff cannot produce a directed edge from patient A to patient B and then a subsequent edge from patient D to patient E without first producing an edge from patient B to patient D).

#### Statistical thresholds for subgraph frequency and uniqueness

A formal definition for network motif candidate detection was given by Ribeiro et al. (2009), derived from the work of Milo et al. (2002), and has become a popular method in this area (Kashani et al. 2009; Patra 2020). Ribeiro and colleagues suggest that an induced size-*k* subgraph $G_{k}$ of a graph G is a network motif when, for a given (self-selected) set of parameters {P, U, D, N} (where P is the probability threshold, U is the uniqueness threshold, D is the proportional threshold, and N is the number of random similar networks), it satisfies three conditions:

- **Condition 1:** Over-representation
  1 $$\begin{aligned} Prob(f_{rand}(G_{k}) > f_{real}(G_{k})) < P \end{aligned}$$
  The number of random networks in which $G_{k}$ appears more than the input network, divided by the number of networks in the random ensemble, where $f_{rand}(G_{k})$ denotes the frequency of the subgraph $G_{k}$ in the random network ensemble, $f_{real}(G_{k})$ denotes the frequency of $G_{k}$ in the network being analysed, and *P* defines the probability threshold. For anti-motifs, the probability that they appear in randomized networks fewer times than in the real network is $P_{anti-motif} < P$, where $P=0.01$ (Milo et al. 2002; Milo et al. 2004b):
  2 $$\begin{aligned} Prob(f_{rand}(G_{k})< f_{real}(G_{k})) < P \end{aligned}$$
- **Condition 2:** Minimum frequency
  3 $$\begin{aligned} f_{\text {real}}(G_k) \ge U \end{aligned}$$
  where $f_{real}(G_{k})$ denotes the frequency of the subgraph $G_{k}$ in the real network and *U* defines the frequency threshold.
- **Condition 3:** Minimum deviation
  4 $$\begin{aligned} f_{real}(G_{k}) - f_{rand}(G_{k}) \ge D \times f_{rand}(G_{k}) \end{aligned}$$
  $f_{real}(G_{k})$ should be significantly larger than $f_{rand}(G_{k})$ to prevent the detection of motifs that have a small difference between these two values but have a narrow distribution in the random networks. *D* is the proportional threshold that ensures the minimum difference between $f_{real}(G_{k})$ and $f_{rand}(G_{k})$. For anti-motifs, there should be a minimum difference between $f_{rand}(G_{k})$ and $f_{real}(G_{k})$, where $D=0.1$ (Milo et al. 2002):
  5 $$\begin{aligned} f_{rand}(G_{k}) - f_{real}(G_{k}) \ge D \times f_{rand}(G_{k}) \end{aligned}$$

There are no widely accepted exact thresholds for these conditions, but it is commonly argued that the more restricted thresholds yield more precise motifs (Kashani et al. 2009). A subtle variation is the notion of an anti-motif Milo et al. (2004a), which is a significantly under-represented subgraph that has also shown to be meaningful (Baskerville and Paczuski 2006; Ashford et al. 2019). We use the same values for *P*, *D*, and *U* as Milo et al. (2002) as a basis for motif and anti-motif candidate selection (Milo et al. 2004b).

#### Motif (and anti-motif) candidate statistical analysis

Typically, after being selected by passing all criteria in Sect. 3.5 original and null frequencies of motif (and anti-motif) subgraphs are subsequently assessed using statistical significance measures such as the *Z-score* (Milo et al. 2002) or *relative abundance* ($\Delta$) (Milo et al. 2004a). Considering the large variation of ward sizes (Table 3) and patient throughput in the study dataset, we apply $\Delta$ as our significance metric for robustness against different network sizes when evaluating the appearances of small subgraphs (Ciriello and Guerra 2008).

6 $$\begin{aligned} \Delta (G_{k}) = \frac{f_{real}(G_{k}) - f_{rand}(G_{k})}{f_{real}(G_{k}) + f_{rand}(G_{k}) + \epsilon } \end{aligned}$$

The error term $\epsilon$ has been shown to work well when set to 3 for triads and 4 for tetrads (Milo et al. 2004a; Felmlee et al. 2021) to prevent the relative abundance approaching infinity in rarely counted subgraphs (Patra 2020). We then calculate the Subgraph Ratio Profile, which is the normalised value of $\Delta$.

7 $$\begin{aligned} SRP(G_{k}) = \frac{\Delta (G_{k})}{\sqrt{\sum \Delta (G_{k})^2}} \end{aligned}$$

#### Subgraph catalogue reduction

As mentioned in Sect. 3.2, there are 217 subgraph variations, with some potentially over- or under-represented in the networks. The literature suggests various methods that reduce the catalogue of subgraphs used for evaluation. Ribeiro (2011) recommends only searching in the null model for subgraphs that appear in the original network to reduce execution time (Shutters et al. 2022), however, in cases where anti-motifs (where subgraph patterns are distinct if dis-proportionally under-represented) are also considered, a complete census must still be performed. Berg and Lässig (2004) also suggests that tetrad variations of triad subgraphs, such as those with a ‘dangling’ edge (i.e., a 3-node subgraph plus one incoming or outgoing edge), can also be excluded from searches to reduce execution time. Other work uses statistical significance metrics to reduce the subgraph catalogue after a full census has been completed, through either selecting the top *n* subgraphs by rank (Milo et al. 2004b) or only discussing subgraphs over a significance threshold (McMillan and Felmlee 2020).

For our results, we reduce our subgraph catalogue using a “Concentration” metric, ($C(G_{k})$), which is determined by how frequently a subgraph $G_{K}$ appears in comparison with other subgraphs of the same size (Milo et al. 2004b). Z-scores for all subgraphs will be presented in the Appendix. If there are *n* number of size-*k* subgraphs in a network, then $C(G_{k_i})$ of the *ith* subgraph $G_{k}$ is defined as:

8 $$\begin{aligned} C_{\text {real}}(G_{k}) = \frac{f_{\text {real}}(G_{k})}{\sum _{i=1}^{n}{f_{\text {real}}(G_{k_i})}} \end{aligned}$$

We exclude 0 values (to two decimal places) for subgraph concentration mean, ($C(G_{k})\mu$), and standard deviation, ($C(G_{k})\sigma$). This accounts for variation in network size and degree when sampling for motifs based on frequency (Ciriello and Guerra 2008).

#### Subgraph ratio profiles

We then use the reduced catalogue of subgraphs to define an SRP for each ward vital signs observations network. This not only presents a clear visual representation of the relative strengths of the local patterns within it, but also allows us to compare against other networks, including those of different types. Here, we conduct an analysis of statistical similarity between the profiles of the different wards using correlation tests similarly to other types of complex networks (e.g., Milo et al. 2004b). The feature vectors will firstly be tested for normality using one-sample Kolmogorov–Smirnov tests to determine whether the correlation should use an appropriate parametric or non-parametric test. Then, dependent on the strength of the correlation results, we use the strength and sign of the correlations to determine whether ward networks are individual, clustered, or form a hospital ward “superfamily” profile. External features, which in our case could be ward size or specialism, have shown to develop clusters in collections of networks within others non-medical domains (Ashford et al. 2019; Tu et al. 2020b) or across domains (Milo et al. 2004a; Shutters et al. 2022).

## Results

We divide our results into three subsections; Network Structure (4.1), Subgraph Analysis (4.2), and Subgraph Ratio Profile Analysis (4.3). We examine network metrics (such as density, closeness, and clustering) across the overall structure of the networks, and categorise observation sequences in the static-temporal construction for a broad overview of the staff behaviour patterns. Then, we describe the concentration and relative frequency of subgraphs. This includes evaluating the execution time required for the frequency analysis, identifying motifs within the network, and assessing the strength of these motifs. This is followed by a comparison of ward SRPs using pairwise test matrices, to determine the extent of any correlation and whether the networks are similarly structured. Finally, we summarise the key findings.

### Network structure

**Fig. 7:**
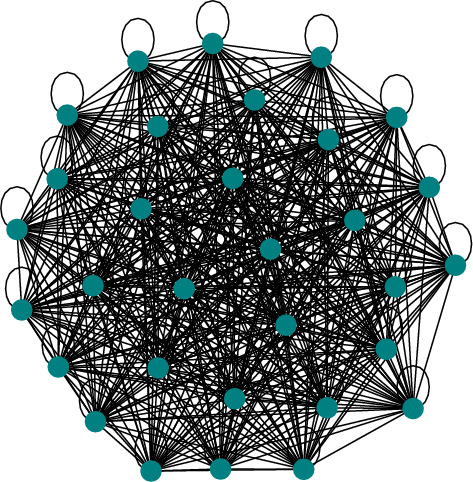
The vital sign observations dataset for W1 visualised as a static network

**Fig. 8:**
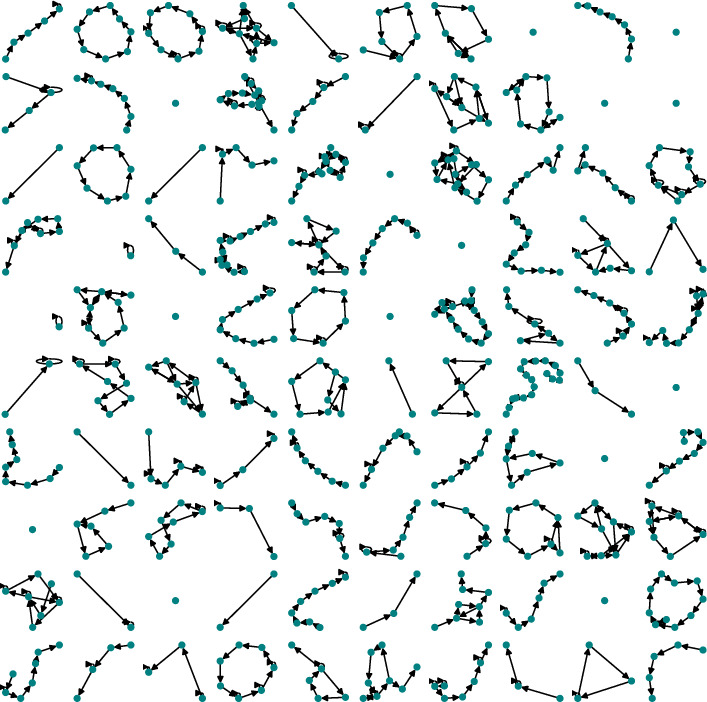
Visualisation of a sample of 100 staff vital sign observation sequence networks extracted from the W1 dataset

Table 4 introduces both network constructions using various metrics (where $N_{edges}$ is the number of edges, $R_{sl}$ is the ratio of self-loop to non-self-loop edges, $\rho _{sl}$ is the density inclusive of self-loops, $\rho$ is network density excluding self-loop edges,^4^$clus_{\mu }$ is the average network clustering coefficient and $clos_{\mu }$ is the average network closeness coefficient). All wards appear to be highly interconnected and tend to form a connected graph when modelled as a static network across extended periods (Fig. 7). High network density with ($\mu =1.02$, $\sigma =0.078$) and without including self-loops ($\mu =0.98$, $\sigma =0.070$), clustering ($\mu =0.99$, $\sigma =0.026$), and closeness ($\mu =0.98$, $\sigma =0.051$), may obscure any nuance in potential behaviour patterns. The static-temporal network is much less interconnected, with low ward network density (with self-loops ($\mu =0.21$, $\sigma =0.067$) and without self-loops ($\mu =0.17$, $\sigma =0.040$)) and closeness ($\mu =0$, $\sigma =0.004$), and negligible clustering ($\mu =0.20$, $\sigma =0.041$). This is a reflection of the expected short and typically simple vital sign observation sequences that make up a clinical staff path during a ward round (see Fig. 8). It is clear that there is general similarity seen across wards and sites, especially in the highly interconnected static network, which will be reflected by highly interconnected subgraphs, such as triad 300 or tetrad T199. On the other hand, the much less interconnected static-temporal network maintains some notable variability that motivates exploring the substructures further.

**Table 4:** Summary of network characteristics for the 20 study wards for both static and static-temporal network representations

	Static network						Static-temporal network					
Ward	$N_{edges}$	$R_{sl}$	$\rho _{sl}$	$\rho$	$clus_{\mu }$	$clos_{\mu }$	$N_{edges}$	$R_{sl}$	$\rho _{sl}$	$\rho$	$clus_{\mu }$	$clos_{\mu }$
W1	44,572	0.1	1.03	1	1	1	35,565	0.6	0.2	0.16	0	0.19
W2	47,532	0.13	1.03	1	1	1	39,506	1.02	0.23	0.18	0.01	0.22
W3	44,836	0.13	1.03	1	1	1	34,064	0.63	0.2	0.15	0	0.18
W4	42,355	0.13	1.03	1	1	1	32,550	0.74	0.2	0.15	0	0.19
W5	54,270	0.16	1.03	1	1	1	43,897	1.08	0.28	0.21	0.02	0.26
W6	45,514	0.23	0.92	0.89	0.91	0.91	33,322	0.98	0.31	0.2	0.01	0.23
W7	37,717	0.1	1.03	1	1	1	33,100	1.03	0.16	0.13	0	0.18
W8	25,301	0.12	1.01	0.98	0.99	0.98	21,185	0.83	0.15	0.12	0	0.15
W9	22,154	0.18	1.06	1	1	1	13,192	0.56	0.42	0.31	0	0.32
W10	25,444	0.13	1.03	1	1	1	20,256	0.7	0.19	0.15	0	0.18
W11	28,500	0.14	1.03	1	1	1	22,956	0.85	0.2	0.15	0	0.18
W12	39,244	0.14	1.03	1	1	1	33,027	1.1	0.22	0.17	0.01	0.22
W13	31,325	0.13	1	0.96	0.98	0.97	27,041	1.04	0.18	0.14	0	0.18
W14	35,202	0.14	1.04	1	1	1	27,332	0.75	0.19	0.15	0	0.18
W15	30,428	0.14	1.04	1	1	1	24,438	0.87	0.27	0.21	0.01	0.26
W16	25,909	0.12	0.71	0.69	0.92	0.79	18,343	0.38	0.24	0.2	0.01	0.24
W17	11,969	0.15	1.06	1	1	1	10,168	0.96	0.15	0.12	0	0.16
W18	28,407	0.14	1.03	1	1	1	23,970	1	0.17	0.13	0	0.17
W19	9293	0.15	1.09	1	1	1	7,505	0.63	0.15	0.13	0	0.16
W20	24,976	0.13	1.03	1	1	1	20,554	0.78	0.16	0.13	0	0.17

Where $N_{edges}$ is the number of edges, $R_{sl}$ is the ratio of self-loop to non-self-loop edges, $\rho _{sl}$ is the density inclusive of self-loops, $\rho$ is network density excluding self-loop edges, $clus_{\mu }$ is the average network clustering coefficient and $clos_{\mu }$ is the average network closeness coefficient

**Fig. 9:**
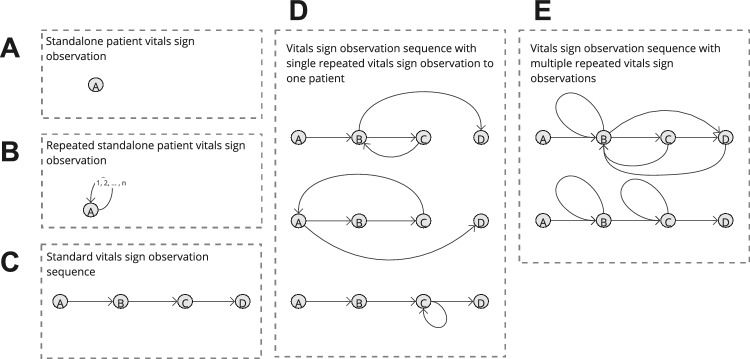
A non-exhaustive example set of vital signs observation sequence networks ($V=4$) defined by 5 broad categories: A, a stand-alone vital sign observation, B, a rapidly repeated stand-alone vital signs observation, C, a vital sign observation sequence, D, a vital sign observation sequence with a single repeated vital sign observation for one patient, and E, a vital sign observation sequence with multiple repeated vital sign observations

The broad structure of routine vital sign observation sequences by clinical staff in the static-temporal model was categorised by the occurrence of a repeated vital sign observation within the sequence, or lack thereof. This exercise provides context to the volume of clinical staff vital sign observation sequences that are complex enough for a variety of network sub-structures to occur. Figure 9 describes five broad observation sequence categories: A, a stand-alone vital signs observation, B, a rapidly repeated stand-alone vital sign observation, C, a vital sign observation sequence, D, a vital sign observation sequence with a single repeated vital sign observation for one patient, and E, a vital sign observation sequence with multiple repeated vital sign observations.

**Table 5:** Summary of routine clinical staff vital sign observation sequences type volumes for each of the 20 study wards

Ward	Total ‘rounds’	A	%	B	%	C	%	D	%	E	%
W1	9008	2409	26.7	1927	21.4	2916	32.4	640	7.1	1116	12.4
W2	8027	1712	21.3	1708	21.3	1850	23	740	9.2	2017	25.1
W3	10,773	3567	33.1	2351	21.8	2623	24.3	896	8.3	1336	12.4
W4	9806	2930	29.9	2358	24	2293	23.4	791	8.1	1434	14.6
W5	10,374	1991	19.2	2225	21.4	2676	25.8	1277	12.3	2205	21.3
W6	12,193	3040	24.9	3244	26.6	2088	17.1	1427	11.7	2394	19.6
W7	4618	645	14	1225	26.5	1249	27	382	8.3	1117	24.2
W8	4117	995	24.2	1092	26.5	1007	24.5	322	7.8	701	17
W9	8963	2444	27.3	1669	18.6	3130	34.9	1191	13.3	529	5.9
W10	5189	1398	26.9	1211	23.3	1472	28.4	445	8.6	663	12.8
W11	5545	1301	23.5	1459	26.3	1369	24.7	514	9.3	902	16.3
W12	6218	950	15.3	1516	24.4	1640	26.4	667	10.7	1445	23.2
W13	4285	724	16.9	1014	23.7	1139	26.6	406	9.5	1002	23.4
W14	7871	2150	27.3	1950	24.8	1859	23.6	800	10.2	1112	14.1
W15	5991	1195	19.9	1216	20.3	1797	30	623	10.4	1160	19.4
W16	7567	2399	31.7	1153	15.2	2914	38.5	527	7	574	7.6
W17	1802	352	19.5	517	28.7	411	22.8	142	7.9	380	21.1
W18	4438	959	21.6	1143	25.8	1061	23.9	394	8.9	881	19.9
W19	1789	490	27.4	434	24.3	527	29.5	120	6.7	218	12.2
W20	4423	1108	25.1	1140	25.8	1155	26.1	369	8.3	651	14.7

Observation sequence type descriptions are illustrated in Fig. 9

About 75–80% of static-temporal ‘snapshots’ are either stand-alone vital sign observations, repeated stand-alone vital sign observations, or sequential vital signs observation sequences (categories A, B, and C in Fig. 9 respectively). None of these categories can be effectively described using subgraph analysis due to their low complexity (see Table 5). Sequences where clinical staff rapidly repeat vital sign observations once to one patient (category D, Fig. 9) represent 7–13% of all vital signs observation sequences, with the rest comprised of high complexity sequences (category E, Fig. 9), which are suspected to represent key ‘non-routine’ ward behaviours. The distinct relative similarity between all wards for vital signs observation sequence type volumes is representative of an abstracted operating policy and/or precedent. However, we also observe a significant spread of D ($\mu =9.18\%$, $\sigma =1.79\%$) and E ($\mu =16.86\%$, $\sigma =5.46\%$) type vital sign observation sequence proportions that is indicative of fluidity in ward operating behaviour.

### Subgraph analysis

#### Motif identification

Table 6 describes the number of wards (*N*) in which a given subgraph meets all three statistical criteria for motif candidacy (Sect. 3.5), truncated by non-zero (to 2 decimal places) values for $C(G_{k})$ $\mu$ and $C(G_{k})$ $\sigma$ values (Figs. 15, 16 show the full concentration profiles for both network constructions). $C(G_{k})$ has been calculated in four separate groups respective to subgraph degree sequence: Nodes and self-loops, dyads, triads, and tetrads. These methods exclude motif and anti-motif candidates with insignificant $C(G_{k})$ scores across all study wards from further investigation, since low appearance volumes cannot be used to calculate statistical significance reliably. The calculated $C(G_{k})$ values reflect the results in Sect. 4.1 and Figs. 7 and 8. The static network sees high counts for completely connected subgraphs (reciprocating dyad, 300, and T198), whereas the primary concentrations for the static-temporal model represent highly disconnected subgraphs that illustrate a chain of observations (012, 021C, and T12).

**Table 6:** Number of wards (N) in which a given subgraph meets motif ($N_{m}$) and anti-motif ($N_{a-m}$) candidate criteria, truncated by non-zero values for subgraph concentration ($C(G_{k})$), mean ($\mu$), and standard deviation ($\sigma$)

	Static				Static-temporal			
Subgraph	$N_{m}$	$N_{a-m}$	$C(G_{k})$ $\mu$	$C(G_{k})$ $\sigma$	$N_{m}$	$N_{a-m}$	$C(G_{k})$ $\mu$	$C(G_{k})$ $\sigma$
Non-self-loop			0.96	0.01		20	0.82	0.05
Self-loop			0.04	0.01	20		0.18	0.05
Dyad (012)		1	0.02	0.05		13	0.98	0.01
Rec. Dyad (102)		1	0.18	0.38	3		0.02	0.01
021D		1				20	0.01	0.01
021U		1				20	0.01	0.01
021C						20	0.93	0.04
111D		2	0.01	0.02	4		0.02	0.01
111U		2	0.01	0.02	4		0.01	0.01
030C	1				1		0.01	0.01
201		1	0.01	0.02				
120C			0.01	0.01				
210		1	0.02	0.05				
300		1	0.95	0.12				
T1		1				20	0.02	0.01
T2						20	0.01	0.01
T5		1			1		0.01	0.01
T11	1					20	0.01	0.01
T12						20	0.86	0.09
T15						20	0.02	0.01
T18	1				1		0.01	0.01
T20					1		0.01	0.01
T34						1	0.01	0.01
T37						1	0.01	0.01
T73							0.01	0.01
T195		1	0.01	0.02				
T197		3	0.01	0.04				
T198		1	0.93	0.17				

Full concentration profiles are shown in Figs. 15 and 16. Both the static and static-temporal network construction results are shown. Values equivalent to 0 to 2 decimal places are blocked out

Subgraphs identified to be motif (or anti-motif) candidates from the criteria detailed in Sect. 3.5 across all or a majority of wards likely reflect routine (or highly irregular) ward behaviours, and subgraphs identified as motif or anti-motif candidates across some wards may represent behaviours relating to a ward characteristic, such as specialisation or site. Considering Table 6, there is low representation for both motif and anti-motif candidates in the static network, showcasing that the most common subgraphs are not necessarily network motifs, and that uniformity seemingly prevents variation in subgraph appearance. Again, in the static-temporal construction the most prominent subgraphs are also still significantly underrepresented in comparison to the null model, however, we successfully identified a set of subgraphs in the static-temporal network likely to reflect heuristic ward-level operation behaviours; 5 that meet motif selection criteria nearly universally in the static-temporal network construction (Self-loops, Reciprocating dyads, 111D, 111U, and 030C) and another 2 tetrads that meet motif selection criteria in about half the wards (T12 and T34). Additionally, we find 7 subgraphs that meet anti-motif criteria in all wards (Non-self-loops, 021D, 021U, 021C, T1, T2, and T11) and note that dyads meet anti-motif criteria in 13 wards. These results are expected as all triad and tetrad anti-motifs have weakly connected components, and it is impossible for them to occur in real-world data.

#### Motif strength evaluation

**Fig. 10:**
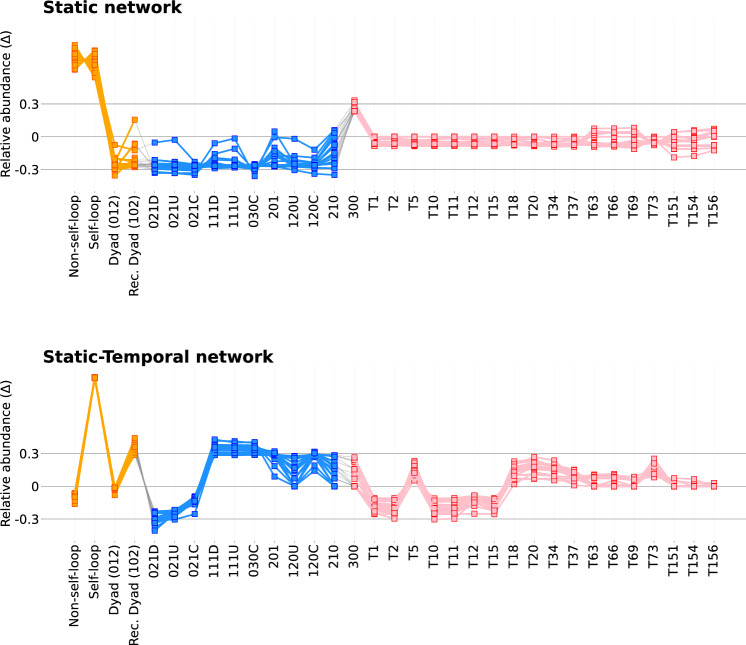
Truncated SRPs of both static and static-temporal network constructions. SRPs are truncated by excluding subgraphs with $C(G_{k})$ $\mu$ values less than 0.01 for their respective construction. Subgraph labels in bold represent subgraphs that pass all motif or anti-motif candidate criteria discussed in Sect. 3.5. Figures 17 and 18 illustrate the non-truncated SRPs for both the static and static-temporal networks

Figures 17 and 18 illustrate SRP profiles for each study ward for both network construction methods, with the truncated version (inclusive of subgraphs with $C(G_{k})$ $\mu$ values greater than 0.01 in either construction, in line with Table 6) shown in Fig. 10. Subgraphs that meet all motif selection criteria in some wards and the threshold for our chosen statistical significance measure, relative abundance ($\Delta$), for which we consider to be 0.3, can be rightly described as network motifs.

Although there are some notable $\Delta$ scores for self-loops (0.99, 0.93, 0.96, 0.99), 102 (0.42), 111D (0.48, 0.56), 111U (0.48), 030C (0.37, 0.50), and 120C (0.43) in the static network SRP profiles, these only occur in a handful of wards, and are without supporting significant $C(G_{k})$ scores. This is likely a reflection of variability in the data rather than evidence of motif or anti-motif presence. Conversely, there are several prevalent subgraphs within the static-temporal network construction that may represent highly regular or highly irregular behaviours. Self-loop edges are the only subgraph to satisfy all three motif selection criteria and exhibit high $\Delta$ scores across all study wards. Whilst not universally meeting all three motif candidate criteria, reciprocating dyads (102), and triads 111D, 111U, and 030C all demonstrate significant $\Delta$ scores (where $\Delta$ is above our threshold value of 0.3 across the majority of wards). It is therefore worth considering setting a precedent to only consider ‘Condition 2: Minimum frequency’ (Ciriello and Guerra 2008; Baskerville and Paczuski 2006; Ashford et al. 2019) for a compelling case in also describing these as network motifs.

Anti-motifs have a broader representation in the preliminary criteria, with 10 subgraphs meeting anti-motif candidacy criteria. Four of these subgraphs (non-self loops, dyads (012), 012C, and T12) have weaker $\Delta$ scores. This represents a notable variation that suggests they are not strictly anti-motifs, and may instead be more reflective of behaviours dependent on ward specialisation. The remaining 6 subgraphs (021D, 021U, T1, T2, T11, and T15) additionally maintain significant $\Delta$ scores and can therefore be regarded as anti-motifs.

**Table 7:** Median (Mdn), mean ($\mu$) and standard deviation ($\sigma$) of Spearman’s correlation coefficients, denoted as *r*, of the static temporal network construction SRP profiles for a selection of subgraph groupings

Subgraph group	$r_{Mdn}$	$r_{\mu }$	$r_{\sigma }$	number of tests with $p <.05$
All subgraphs	0.760	0.715	0.147	190
self-loops & dyads	1.000	1.000	0.000	190
triads	0.957	0.941	0.049	190
tetrads	0.692	0.630	0.202	176
self-loops, dyads & triads	0.968	0.962	0.025	190

Median, mean, and standard deviation values are calculated from all ward comparison tests ($N=190$). Figures 19 and 20 show the full pairwise test matrices for the static and static-temporal network construction, respectively

### Comparing subgraph ratio profiles between wards

One-sample Kolmogorov–Smirnov tests for normality confirmed that in both constructions all distributions of subgraph ratio profiles were not normally distributed (results are described in Table 10, which in all cases reject the null hypothesis). Spearman’s Rank-Order Correlation tests were therefore used to complete pairwise tests between wards. Additionally, a False Discovery Rate (FDR) correction was applied to account for type I errors. The results are summarised in Table 7 and full correlation coefficient matrices are shown in the Appendix (Figs. 19, 20). The results support the visual similarity observed between static-temporal ward network SRPs in Fig. 10. The correlation tests were also significant in all results for low-order subgraphs, triads, and for the combined subgraph catalogue. While correlation tests for tetrads weren’t significant in every case, they were also still significant in most (in 176 of 190 tests).

These results strongly imply the existence of a higher-level classification beyond individual networks based on the overarching design principles governing the general intra-patient ward operational strategy. Overall, the results reaffirm the similarities between wards, as evidenced by the consistent vital signs observation sequences categories (Table 5), and indicate that this level of detail is likely to be insufficient to detect influences from features such as specialism, size, and hospital layout using clustering or graph embedding methods as seen in studies examining other types of complex networks (Ashford et al. 2019; Tu et al. 2020b).

**Table 8:** The mean, $\mu$, and the standard deviation, $\sigma$, of the execution time to complete a census of subgraph frequencies for a ward on non-specialised computing equipment

Network construction	Subgraph census	Runtime $\mu$ (s)	Runtime $\sigma$ (s)
Static	Self-loops, Dyads, and Triads	0.008	0.04
	Tetrads	0.019	0.01
	All	0.027	0.014
Static-temporal	Self-loops, Dyads, and Triads	0.241	0.091
	Tetrads	0.037	0.019
	All	0.279	0.108

#### Execution time for different scenarios

Typical execution times^5^ to complete different censuses for the networks of each ward are summarised in Table 8. A complete census was achieved in under 1 s for all scenarios, i.e., irrespective of the network construction, the types of subgraphs to include, and the ward. Network frequency censuses were also repeated for equal sized random networks (see Sect. 3.5), resulting in similar execution times. The number of random networks used may vary depending on the specific applied scenario by a stakeholder (Ribeiro and Silva 2010), therefore the total execution time for modelling a ward can therefore be estimated as $T_{real\ census} + (N_{random\ networks}* T_{real\ census})$ (where *T*=the time taken and *N*=the number of networks). After the subgraph frequency counting stage, each network is described as a 1 by 217 dimensional vector, and all subsequent calculations are then the same irrespective of the scenario.

A breakdown for different scenarios by ward, network representation type, and subgraph types is shown in Table 9 (Appendix). The ward characteristics in this dataset represent a range of different hospitals, ward specialisms, number of beds, and number of staff (Table 3), which are representative of hospital wards in general (Giancotti et al. 2017). The execution times are similar for wards with different non-network based characteristics (e.g., ward type, number of observations, or specialism, as outlined in Table 3) across both network representations and different subgraph types.

However, there are notable, relative differences between different specific scenarios that may be attributable to network characteristics. For example, for scenarios where a static-temporal network representation is used, the subgraph frequency analysis takes longer than the static network in all cases where all subgraph types are used, and almost all cases where subset of subgraph types are used (e.g. W19, tetrads only as an exception). This is despite the static-temporal network representations having a smaller number of edges (as shown in Table 4) than the static network representation.

A potential reason for this is the differences in density between the static network and static-temporal representations in this dataset, where very dense networks may have an abundance of a limited number of specific tetrad subgraphs that are quicker to determine and count. This presents some additional considerations where the methodology may be applied to new hospital ward data, or datasets and networks representing other types of behaviour or complex systems.

### Key results summary

The four key results described in Fig. 1 can be summarised as follows:

**Fig. 11:**
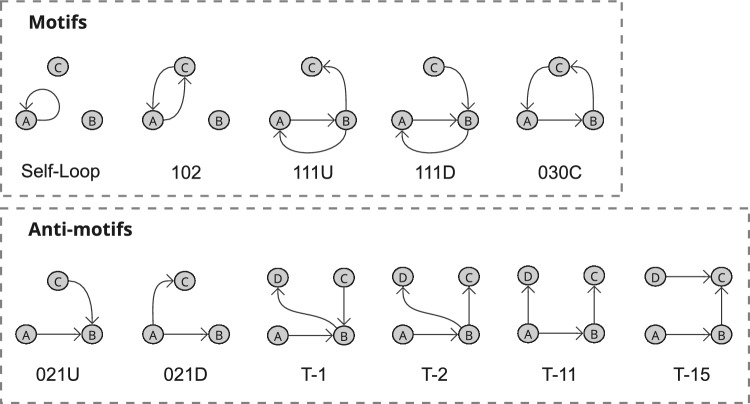
Hospital ward motifs (Self-Loop, 102, 111U, 111D, and 030C) and anti-motifs (021U, 021D, T1, T2, T11, and T15) in the static-temporal network construction of vitals observation recordings (Sect. 4.4). Motifs and anti-motifs have been identified based on performance against criteria described in Sect. 3.5 and relative abundance, $\Delta$, scores

**1**: **Uniformity in the static network representation motivates using time as a network element.** Figures 7 and 8, and Table 4 demonstrate that when evaluating all observations without making adjustments for staff ID or period, the static network tends toward a complete graph and loses information.
**2**: **Staff vital signs observation sequences typically lack subgraph complexity.** Most staff members vital signs observation sequences are either linear sequences or supplementary stand-alone vital signs observations (Table 5).
**3a**: **We define 5 motif patterns in staff vital signs observation sequences: Self-loops, reciprocating dyads (102), and triads (111D, 111U, and 030C).** Illustrated in Fig. 11, the identified highly regular network subgraphs demonstrate the processes of clinical staff returning to a previous patient within short timeframes.
**3b**: **We define 6 anti-motif patterns in staff vital signs observation sequences: 021D, 021U, T1, T2, T11, and T15.** Illustrated in Fig. 11, the identified highly irregular network subgraphs demonstrate movement patterns that are not possible to occur when considering the recording behaviour of individual staff members, and therefore are not significant.
**4**: **External ward characteristics have little impact on rates of specific staff behaviours for routine vital signs observations.** Closeness in network structure measures (Tables 4 and 5) and local substructures (Fig. 10 and Table 7) in the static-temporal representation show wards produce similar relative rates of specific staff movement patterns despite differences in ward size, specialism, staffing levels, and architecture.

## Discussion

The two network representations in this study, static and static-temporal (Figs. 7, 8), have demonstrated markedly different results. When incorporating all vital signs observations over the complete 12-month period dataset for each ward into a static network, we note that the network progresses toward a complete graph (Result 1, Fig. 1). This is unlike other applied scenarios, such as airline networks, where journeys are typically restricted to the terminals for which a company has paid for the route. In this case, it may signify the flexibility in the allocation of beds where clinical staff complete their vital sign observations. This result contributes to RQ1 by motivating static-temporal network representations of a vital sign observations using sequences that include time and staff elements in order to support higher specificity studies, such as describing the patterns that occur when clinical staff rapidly repeat routine patient vital signs observations.

In the static-temporal network, all wards saw significant volumes and $C(G_{k})$ scores of 012C and T12 subgraphs and high proportions of type A (stand-alone), type B (repeated stand-alone observation), and type C (sequential without patient return actions) vital sign observation sequences (Table 5). This may suggest a managerial precedent to complete ward rounds as a simple vital sign observation sequence that include all patients within an individual clinical staff member’s scope-partially addressing RQ2. This would still allow for the escalation of patient care responsibility of serious adverse events (such as high NEWS readings) to senior or tasked members of staff accordingly. Yet, there is a significant fluidity in which clinical staff undertake and record vital signs observations within ward rounds, as demonstrated by the occurrence of types D and E observation sequences. The most common subgraphs 012C and T12 (which represent simple sequences of vital signs observations) both meet anti-motif criteria and maintain significantly low $\Delta$ scores when measured against a BDE null model. Moreover, we see high representation of immediate repetition of vital sign observations behaviours reflected in the identified network motifs (see Fig. 11)-contributing further to RQ2.

A clinical staff member may rapidly repeat a vital sign observation for different reasons. For instance, this may be reflective of patients that are on the threshold of a ‘high NEWS’ score (where NEWS is considered high when it exceeds a score of 5 in total or 3 in one vital sign, RCP (2012a)). Alternatively, it may simply be a low stakes route alteration caused for instance by a patient fall, a patient away from their bed, overseeing a student observation, or monitoring short-term medication effects. These examples highlight the opportunity our method provides to model a collection of vital signs observation recordings as separate sequences which can support further research into the drivers of these repeat observations during specific periods of regular ward activities, like rounds and shift handovers. Additional vital sign observation information, like NEWS and staff concern observation labelling (i.e., ’is concerned’ described in Sect. 3.1 and Table 2), may also be useful in interpreting the role of motifs within specific situational contexts like ward rounds and shift handovers.

**Fig. 12:**
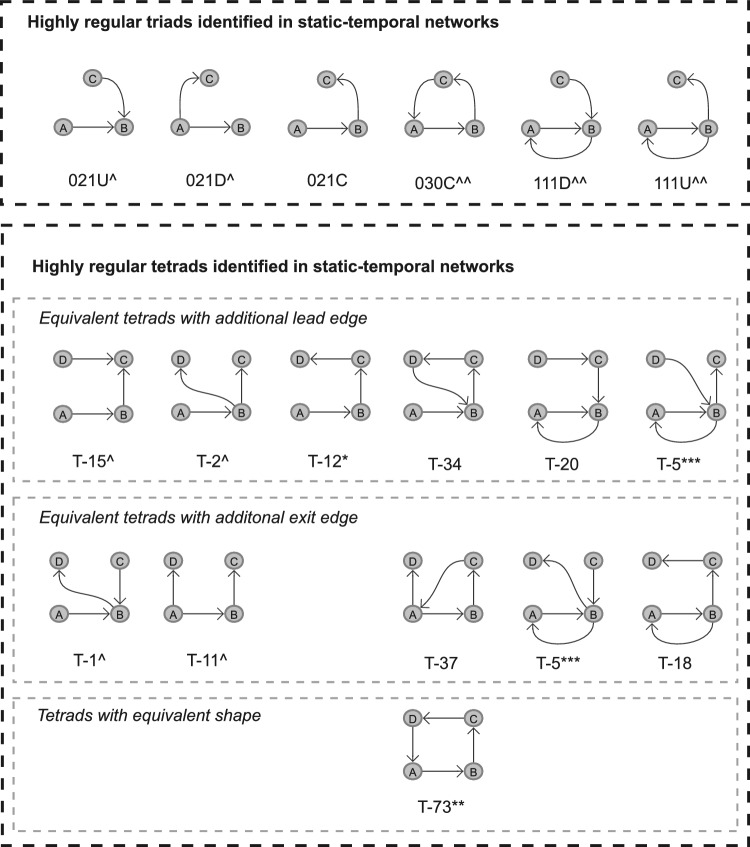
Significant tetrads identified in the static-temporal vital sign observation networks effectively mirror the identified significant triads, accompanied by an additional leading or exiting edge. Where $\wedge$ represents anti-motifs, $\wedge \wedge$ motifs, * tetrads T12 that demonstrates both a lead and exit edge on top of a triad 021C, ** tetrad T73, the equivalent shape of 030C, and *** tetrad T5 which occurs twice

The importance of including tetrads when defining an SRP profile for ward vital sign observation data was less significant than expected. In this study, we suspected that ward specialism, architectural artefacts (i.e., wards segregated into smaller rooms of 2, 3, or 4+ beds), or local patient flow management practices, may present alternative patterns when considering tetrads in comparison to smaller subgraph types (e.g., dyads or triads). However, all highly represented tetrads appear to match highly represented triad subgraphs in the static-temporal network, just with an additional leading or exiting edge (illustrated in Fig. 12). In this somewhat common case (Milo et al. 2004a), it is reasonable to suggest as an additional consideration for RQ1 that tetrads in vital sign observation networks reflect the same behavioural traits as triads and could be excluded in subsequent studies where execution environments have limited computationally capability.

Furthermore, we repeatedly highlight the distinct relative similarity in which wards operate in all the intra-ward comparisons throughout this study-addressing RQ3. The significant correlations in observation sequence categories distributions (Result 2, Fig. 1) and SRP profiles (Table 7) present reasonable evidence that the aggregation of vital sign observation sequences in different wards results in similar frequencies of network sub-structures (Result 3, Fig. 1), and therefore arguably conform to “superfamily” behaviour (Milo et al. 2004a; Result 4, Fig. 1). We expected some similarity due to ward adherence to common health board policies (e.g., ABUHB (2017)), but the high level of similarity is worth highlighting given the variety in ward specialisms, staff, ward layouts, and geographical sites. The profile also appears unique to routine ward vital sign observation networks when compared to other works evaluating World Wide Web hyperlink, biological, energy trade, and social networks (Milo et al. 2004a; Shutters et al. 2022), which typically show much lower significance for subgraphs with strongly connected components (such as motifs 111D, 111U, and 030C). This similarity also presents considerations for future graph embedding tasks (e.g., Tu et al. (2020b)) where effective network identification using subgraph profiles may be limited (contributing to RQ1).

Additionally, despite the quick runtime (Sect. 4.3.1) the high similarity among the ward SRP profiles presents opportunities for feature reduction where necessary in the execution environment. High *r* values for all subgraph groupings (see Table 7) highlight the potential to reduce the SRP feature vector to a subset of subgraph types. This also contributes to RQ1 by having the obvious benefit of reduced computational requirements, while also enabling broader comparison with other data and networks (e.g. (Milo et al. 2004a; Shutters et al. 2022)) where required that may, for example, not have self-loops.

### Clinical implications and applications

While the scope of this study surrounds basic research in modelling and examining human behaviour in hospital ward care settings, the findings and contributions present implications for clinical practice through the identified similar motifs and anti-motifs across the wards. It also provides additional considerations for specific, applied use cases of the framework. Different hospital stakeholders, from staff on wards, to managers on wards and sites, to health boards and trusts, can benefit from summarisations of how activities on wards are undertaken. The re-purposing of data from key, routine ward activities surrounding patient care in vital sign observations can provide a basis for this without the practical challenges and costs of bespoke equipment or third-party observers.

The modelling and analysis framework used here can provide the foundation for the development of future tools that summarise and visualise ward behaviour, alongside other data relevant to the stakeholder and use case (e.g., alongside timeliness, patient outcomes, or other factors highlighted in complementary studies (Sect. 2.2)). For example, hospital managers or policymakers may undertake retrospective reviews of ward activity, either routinely, as a result of a staffing policy, training change, or as a result of a disruptive event, where the results provide a baseline of typical ward behaviour. Any deviations thereof could bes identified in the network and SRP profiles over the time period in question. Additionally, this could and help contextualise instances where staff immediately repeat an observation, or repeat an observation within the same sequence, to identify and manage patients that require additional attention, even if they are not presenting threshold NEWS scores. These examples are not exhaustive, and the network representation may also provide utility in supporting other clinical activities, such as summarising the state of ward activity during shift handovers for staff on wards. Any new or changes to policies or staff resource allocations built upon this information may then impact on other relevant aspects to the use case, such as the timeliness and compliance of vital signs observations.

## Limitations and future work

The dataset used in this study represents vital sign observations of the period of a year. However, the accuracy of vital sign observations is dependent on the point at which they are taken, and adherence to regular and timely recordings remains variable as observed in the literature (Sect. 2.2). While the volume of observations and the observation sequence category distributions (Sect. 4.1) are as expected (Sect. 3.1), there is potential for some data gaps where, for example, a patient is escalated to a doctor and the patient is under constant supervision, that observations are no longer documented on the devices and may instead be taken using pens and paper (Yeung et al. 2012). There may also be unknown cases and data gaps where observations were undertaken on paper where no device was available (e.g., due to being used or due to low battery, for example). The effect of this is mitigated here through the aggregation of data over a large time period, however this presents considerations for future studies and applications, where a further mitigating strategy may be to focus on ‘routine’ observations (i.e., those taken in, or planned for, ward rounds) and their compliance.

Furthermore, the focus of this study has been to present the framework and typical observed behaviours over a large time period. Future studies, or applied use cases, may wish to undertake the modelling and analysis over shorter time periods to observe any changes to the identified motifs, anti-motifs or overall SRPs around potentially severely disruptive events (e.g., sepsis onset) or a less consequential event (e.g., student training observations). Further work could also explore how ward conditions (namely current patient NEWS and TTNOs) leading to the emergence of the prominent self-loop and triad motif subgraphs (111D, 111U, and 030C) that are reflective of clinical staff rapidly repeating vital sign observations (i.e., disruption to ‘typical’ behaviour flows), fluctuates before and after their occurrence. The context behind routine ward rounds behaviour, including when, why, and to what extent disruption occurs.

### Future applications with machine learning

In Sects. 2.3 and 3.3, we discussed the use of machine learning for approximate network motif mining, noting that although the primary advantage is speed, given the context of the study and the network sizes, the drawback of approximation motivates exact subgraph counting and motif extraction in this domain. However, the methodology here is flexible for future applications involving significantly larger networks, such as health board/trust or governments scaling across all hospitals in their remit. Additionally, the SRP profiles could serve as feature vectors for graph embedding tasks (e.g., Tu et al. (2020b)), or in other downstream applied machine learning tasks such as the prediction of related, external factors such as ward observation timeliness, or other compliance values. The applicability and utility of these tasks will depend on the needs of specific stakeholders (Sect. 5.1).

## Conclusions

Understanding how hospital wards undertake routine tasks such as vital signs observations can be a valuable basis for supporting decision-making in patient care management. In this study, we explore a large dataset of anonymised vital sign observations and observe key characteristics and behaviours through the development and use of a network modelling and analysis framework.

The framework has a number of advantages and disadvantages. The primary advantages of this framework are its flexibility in being generally agnostic to specific software, requiring a limited number of expected data fields (Table 2), and time sorted observations. It is also flexible to the type of network representation used and the types of subgraphs considered. In this study, we show the use of multiple different sets of subgraph types together, spanning from self-loops to tetrads. A disadvantage, or note of caution for the framework is the potential for the extracted motifs, anti-motifs and profile to be influenced by the size of the network forming the input data. For instance, hospital wards with a handful of beds (such as in some small community hospitals) may not produce a distinct profile or motifs relative to random networks.

Using the framework, we also provide several additional contributions highlighted in the results and discussion (Sect. 5) that provide insights and recommendations from the dataset used. For example, we show that the inclusion of temporal data in the network construction yielded additional utility for motif extraction and profiling, with a small increase in real execution time. This may limit the ability to scale to significantly larger networks, but if necessary a reduction in the types of subgraph could be used, or subgraph/motif approximation techniques could be adopted, with the results from the exact count here used for reference to help mitigate any potential issues with approximation accuracy.

Overall, we find that modelling the data as a static-temporal network and employing statistical criteria to identify highly regular and irregular subgraphs is shown to be effective in encapsulating typical behaviours surrounding vital sign observations that occur within wards. Namely, that routine patient vitals observations are often executed as simple sequences supplemented by stand-alone observations when repeat observations are required. Additionally, we find that ward size, hospital site, and specialisms do not create notably different behaviours and that the similar, distinct profile aligns with the presence of “superfamilies” of complex networks in other domains observed in similar works (Milo et al. 2004b; Felmlee et al. 2021; Turner et al. 2019).

## Figures and Tables

**Fig. 13:**
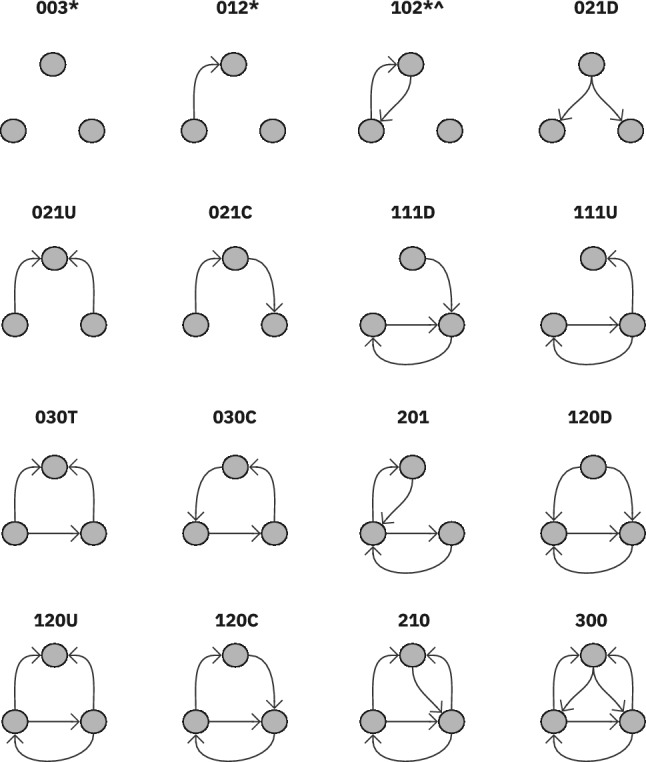
Variations of both static directed dyads, 012 and 102 (sometimes described as ‘Reciprocating Dyad’), and all 13 triad subgraphs. * marks weakly connected subgraphs (where any node in a subgraph cannot be reached by any other node) and $\wedge$ marks subgraphs with strongly connected components (where there is a path between all vertices in the subgraph). Triads are labelled in line with convention (Batagelj and Mrvar 2001)

**Fig. 14:**
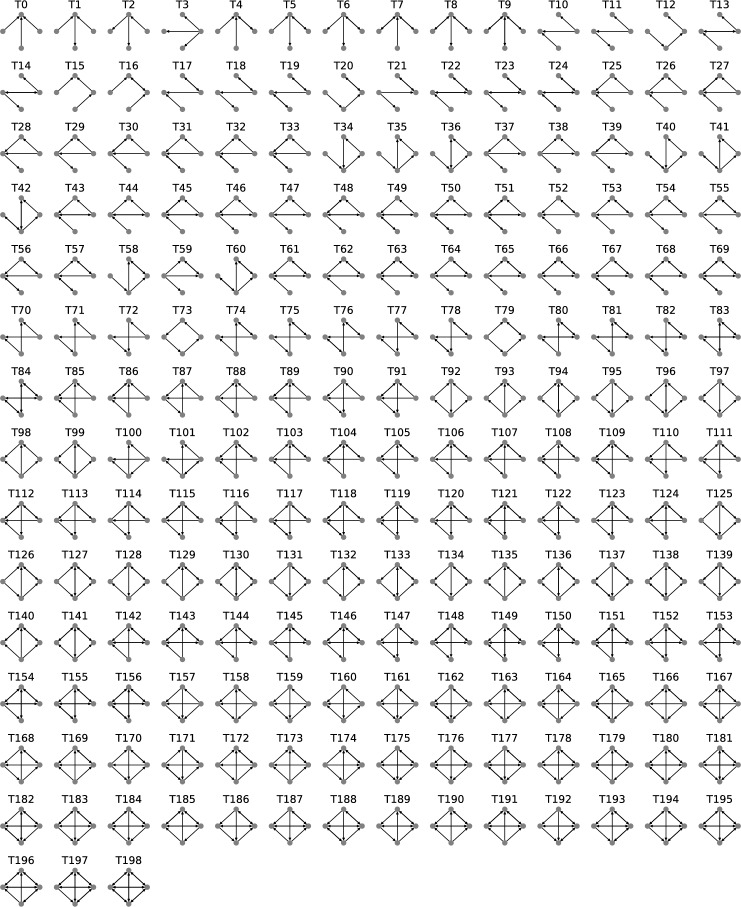
All 199 tetrads with labels

**Fig. 15:**
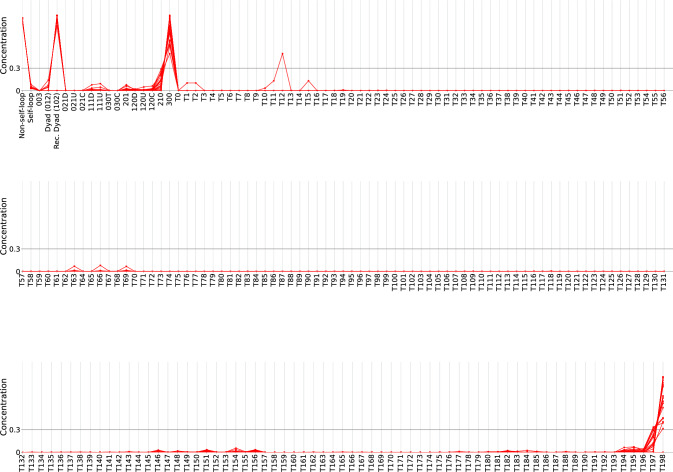
Concentration profile for all 217 subgraphs in the static network construction

**Fig. 16:**
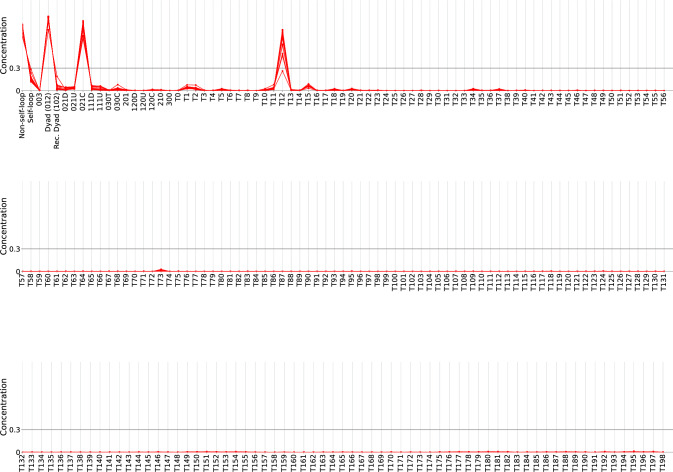
Concentration profile for all 217 subgraphs for static-temporal network construction

**Fig. 17:**
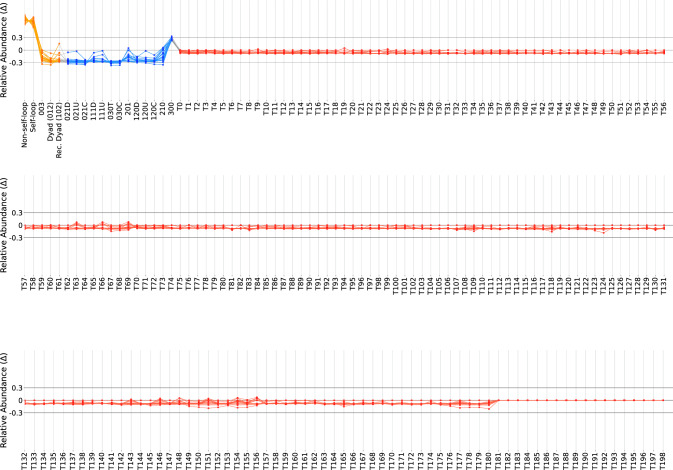
Non-truncated SRP for all subgraphs for the static network construction

**Fig. 18:**
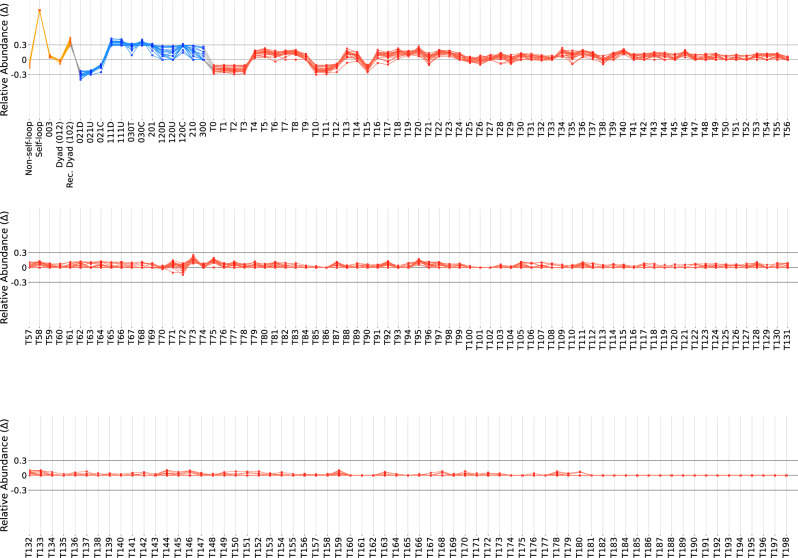
Non-truncated SRP for all subgraphs for the static-temporal network construction

**Fig. 19:**
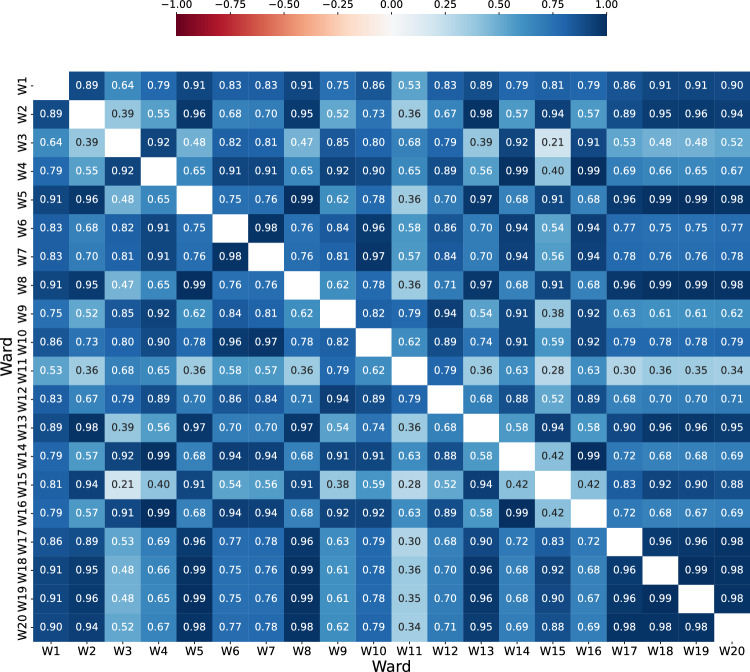
Spearman’s correlation pairwise test matrix for the static network ward representation

**Fig. 20:**
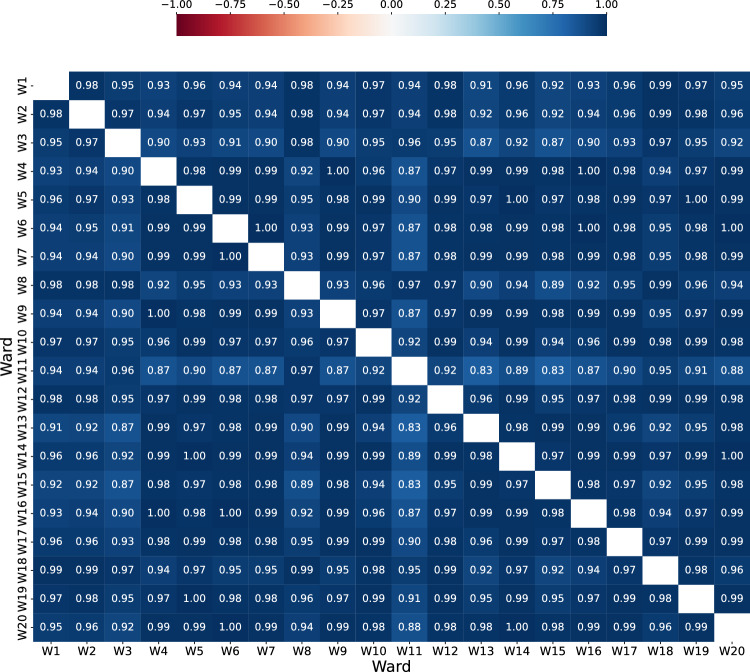
Spearman’s correlation pairwise test matrix for the static-temporal network ward representation

## Data Availability

The dataset used in this study was provided by Aneurin Bevan University Health Board and not available for public release by the authors due to its sensitive context. Aneurin Bevan University Health Board should be contacted for access.

## Appendix: Tables

See Tables 9, 10 and 11.

**Table 9:** Execution runtimes to generate a subgraph census for all wards and for all network construction scenarios

	Static network			Static-temporal network		
Ward	Self-loops, dyads, triads	Tetrads	All subgraphs	Self-loops, dyads, triads	Tetrads	All subgraphs
W1	0.012	0.001	0.013	0.315	0.096	0.411
W2	0.002	0.003	0.005	0.162	0.02	0.182
W3	0.01	0.023	0.032	0.277	0.045	0.322
W4	0.011	0.029	0.04	0.228	0.025	0.253
W5	0.004	0.01	0.014	0.289	0.038	0.327
W6	0.008	0.023	0.031	0.262	0.038	0.3
W7	0.009	0.022	0.031	0.256	0.028	0.284
W8	0.007	0.017	0.023	0.175	0.03	0.205
W9	0.01	0.028	0.038	0.212	0.025	0.237
W10	0.01	0.022	0.032	0.322	0.071	0.393
W11	0.01	0.02	0.03	0.166	0.027	0.193
W12	0.005	0.012	0.017	0.197	0.031	0.227
W13	0.002	0.004	0.006	0.08	0.013	0.093
W14	0.013	0.036	0.049	0.174	0.029	0.203
W15	0.001	0.001	0.002	0.054	0.009	0.063
W16	0.009	0.023	0.032	0.152	0.024	0.176
W17	0.012	0.028	0.04	0.335	0.093	0.428
W18	0.009	0.022	0.03	0.347	0.052	0.399
W19	0.011	0.094	0.105	0.317	0.048	0.366
W20	0.011	0.027	0.038	0.324	0.044	0.367

Censuses for self-loops, dyads and triads were generated with NetworkX Triadic Census and the tetrad censuses were generated with GTrieScanner

**Table 10:** P value results from a one-sample Kolmogorov–Smirnov test for normality, applied to the SRP feature vectors determined in this study, indicate that in all ward cases and for both network construction methods, the SRP is not likely to be normally distributed

Ward	Static	Static-temporal
W1	0.000017	0.000021
W2	0.000017	0.000100
W3	0.000084	0.000025
W4	0.000059	0.000145
W5	0.000010	0.000098
W6	0.000049	0.000092
W7	0.000048	0.000111
W8	0.000006	0.000028
W9	0.000049	0.000176
W10	0.000036	0.000059
W11	0.000257	0.000019
W12	0.000054	0.000062
W13	0.000017	0.000168
W14	0.000018	0.000102
W15	0.000017	0.000395
W16	0.000054	0.000151
W17	0.000005	0.000072
W18	0.000006	0.000023
W19	0.000004	0.000079
W20	0.000004	0.000157

**Table 11:** Summary of longitudinal retrospective observational studies that assess the frequency and compliance of managing vital signs observations

Study	Dataset	Study setting and period	Purpose	Results
Armstrong et al. (2008)	400 vital signs observations (medical records)	Adult patients from “major” or “resuscitation” areas of a district general hospital emergency department in England	To assess the frequency and compliance of vital signs observations	Recording of vital signs was poor and unrelated to staffing levels or numbers of patients attending the emergency department. There was a significant relationship between the failure to record vital signs and lower triage categories
McGain et al. (2008)	211 vital signs observations (medical records)	Adult patients following major surgery in five Australian hospitals between August 2003 and July 2005	To assess the frequency and compliance of postoperative vital signs observations	Hospital and patient factors are associated with incomplete documentation of clinical review and vital signs after major surgery
Leuvan and Mitchell (2008)	1597 vital signs observations (medical records)	2 wards within a tertiary hospital across a 48-hour period in October 2005	To assess the frequency of vital signs observations	Blood pressure, heart rate and temperature were the most diligently recorded vital signs, but documentation of respiratory rate was poor
Hands et al. (2013)	950,043 vital signs observations (VitalPAC)	All patients within the Portsmouth Hospitals NHS Trust, England, between May 2010 and April 2011	To assess the frequency and compliance of vital signs observations	The daily pattern of observation documentation was identical on all days, but not uniform; there were large morning and evening peaks, and lower nighttime documentation. Sicker patients appear more likely to have vital signs measured overnight, but their observations were often not followed by timely repeat assessments
Watson et al. (2014)	Medical records	7 non-intensive care units in an academic paediatric medical centre in a large metropolitan area over 2 time periods; four 12-h day and four 12-h night shifts, approximately 6 months apart	To assess compliance and workflow constraints that exist for documenting vital signs observations and EWS calculations	Significant delays and inconsistencies in vital signs documentation and EWS calculations. Staff felt ambivalent about EWS effectiveness that they had not collected themselves, and were frustrated by the time and pressure involved in scoring and documentation
Johnson et al. (2014)	202 vital signs observations (medical records)	Emergency department within an urban, midwestern United States, teaching hospital	To assess the frequency of vital signss observations	Lower patient acuity was the strongest predictor of increased time between vital signs observations out of personal health factors
Miltner et al. (2014)	(electronic health record)	Emergency department within Veterans Health Administration facilities for 12 randomly selected days in 2011	To assess the frequency and compliance of vital signs observations	A lack of consistent process in documentation of vital signs may decrease the care team’s ability to note early warning signs of physiological instability or deterioration
van Galen et al. (2016)	477 vital signs observations (medical records)	Unplanned ICU admissions from general wards in the VU University Medical Center in Amsterdam, Netherlands, in 2015	To assess the root causes of unplanned ICU-admissions and determine adherence to a Track and Trigger system	Almost half of unplanned ICU admissions from the general ward had healthcare worker related root causes, mostly due to monitoring failures in clinically deteriorating patients
Gale-Grant and Quist (2018)	4775 vital signs observations recordings (Live Obs). Survey data for staff and service users was returned anonymously via paper forms	Two wards; a 22-bed male adult acute inpatient unit, and a 10-bed male psychiatric intensive care unit, across two hospital sites within the same NHS Trust over a period of 10 months	To assess the implementation and feasibility of electronic vital signs monitoring	Nursing compliance improved significantly and users reported positively to the new system
Griffiths et al. (2018)	3,702,717 vital signs observations (VitalPAC). Patient and nurse staffing data was obtained from a patient administration system and electronic rostering systems	32 wards in an acute care NHS hospital, England, between April 2012 and March 2015	To investigate if low nurse staffing levels lead to more adverse patient outcomes and whether missed vital signs observations play a mediating role	Higher nurse staffing levels were associated with fewer missed observations, reduced length of stay and less adverse events, including mortality
Redfern et al. (2019)	2,864,975 vital signs observations (VitalPAC). Patient and nurse staffing data was obtained from a patient administration system and electronic rostering systems	32 wards in an acute care NHS hospital, England, between April 2012 and March 2015	To demonstrate an association between nurse staffing levels and an objective measure of complete and timely care in relation to monitoring patients’ vital signs	Compliance with vital signs monitoring schedules is sensitive to levels of RN and NA staff, however, substantial increases in numbers of staff would be required to effect meaningful increase in adherence. It is likely that other factors, such as clinical judgement, are the main drivers of non-adherence
Dall’Ora et al. (2019)	Same data set as Redfern et al. 2019	32 wards in an acute care NHS hospital, England, between April 2012 and March 2015	To assess the frequency and compliance of vital signs observations for nurses working long shifts	On days when a higher proportion of hours worked by healthcare assistants are from long shifts, the risk of delaying vital signs observations is higher, suggesting lower job performance
Redfern et al. (2019)	538,238 nursing shifts taken over 30,982 ward days	32 medical and surgical wards in an acute general hospital in England	To assess the relationship between staffing levels and vital signs observations compliance	Vital signs observation compliance is sensitive to levels of staffing, although high acuity observations appeared unaffected by levels of nursing assistants
Kostakis et al. (2021)	VitalPAC	Portsmouth Hospitals University NHS Trust between January 2018 and April 2020	To assess the frequency and compliance of vital signs observations between a control period and periods burdened by Coronavirus disease 2019 (COVID-19)	More daily observation sets were performed during the COVID-19 era than in the control periods. Otherwise, COVID-19 did not affect the pattern or compliance of vital signs observations
Eddahchouri et al. (2021)	1,663,743 vital signs measurements over 48,864 patients admitted to general wards between 2015 and 2018	Wards within a tertiary referral university medical centre	To assess the frequency and compliance of vital signs observations and EWS calculations	EWS assessments were incomplete in one-quarter of measurements, and compliance to a MEWS safety protocol was generally low. More MEWSs were recorded in patients with adverse events, however, the increase in vital signs measurements’ frequency mostly occurred shortly before the event manifested. This finding suggests missed opportunities to detect clinical deterioration
Noë et al. (2022)	244,131 vital signs observation (CareFlow, formerly VitalPAC)	8 wards within two Aneurin Bevan University Health Board (ABUHB) hospitals, Wales, for a 12-month period	To assess the frequency and compliance of vital signs observations between ward sizes and specialisms	Ward type and the distribution of patient observation intervals within a ward are significant predictors of temporal observation patterns. Observation patterns also showed evidence of grouping of routine observation recordings
Kim and Jin (2022)	Vital signs measurements at 1-minute resolution (IntelliVue) for 25,751 patients	Continuously monitored beds in the Stanford Health Care Emergency Department between August 2020 and December 2021	To develop measures of fit between documented and actual patient vital signs throughout the visit, as determined from continuous physiologic monitoring, and to compare the performance of actual practice with alternative patient monitoring strategies	Actual documentation of emergency department vital signs observations was variable and incomplete. Alternative monitoring strategies may improve on current practice without increasing the overall frequency of patient monitoring
Jackson et al. (2023)	4,375,654 vital signs observations (electronic health record)	Oxford University Hospitals, England, between January 2016 and June 2019	To assess the frequency and compliance of vital signs observations in relation to associated patient and hospital factors	Vital signs were not always be accurately documented, and this varied by patient groups and hospital settings

## Figures

See Figs. 13, 14, 15, 16, 17, 18, 19, 20.

## Footnotes

1 CareFlow eObservations is a software product from SystemC, formerly known as VitalPAC, as part of a broader Electronic Patient Record (EPR) package (SystemC 2017, 2023).

2 Triadic census is a tool within the NetworkX Python library. Software and user’s guide available at https://networkx.org/documentation.html.

3 gTrieScanner (Ribeiro 2014) is a software that uses the g-trie data structure to count occurrences of induced subgraphs according to their isomorphic class (McKay 1981; Patra 2020) within larger network graphs (software and users guide available at https://www.dcc.fc.up.pt/gtries/output).

4 In networks where self loops are counted in the total number of edges graphs can have density higher than 1.

5 All tests were executed on an 2022 Apple MacBook Pro, with the M2 processor and 16 GB RAM.
